# Specific subsets of urothelial bladder carcinoma infiltrating T cells associated with poor prognosis

**DOI:** 10.1038/s41598-023-39208-0

**Published:** 2023-08-07

**Authors:** Rui Guo, Luyao Wang, Suhang Bai, Danyue Kang, Wei Zhang, Zhenshan Ding, Tianying Xing, Mingxuan Hao, Youfeng Liang, Binbin Jiao, Guan Zhang, Lu Ying, Ruolan Chen, Xiaoyang Chen, Wenjing Zhang, Jiansong Wang, Chuanxing Wan, Changyuan Yu, Haifeng Wang, Zhao Yang

**Affiliations:** 1 College of Life Science and Technology, Innovation Center of Molecular Diagnostics, Beijing University of Chemical Technology, Beijing, 100029 China; 2 College of Life Science and Technology, Key Laboratory of Protection and Utilization of Biological Resources in Tarim Basin of Xinjiang Production and Construction Corps, Tarim University, Alar, 843300 Xinjiang China; 3 College of Veterinary Medicine, The Ohio State University, Columbus, OH 43210 USA; 4 Department of Urology, The Affiliated Hospital of Hebei University, Baoding, 071030 China; 5 Department of Urology, China-Japan Friendship Hospital, Beijing, 100029 China; 6 Department of Urology, Xuanwu Hospital, Capital Medical University, Beijing, 100053 China; 7 Department of Urology, The Second Affliated Hospital of Kunming Medical University, Kunming, 650101 China

**Keywords:** Tumour biomarkers, Urological cancer

## Abstract

Comprehensive investigation of tumor-infiltrating lymphocytes in cancer is crucial to explore the effective immunotherapies, but the composition of infiltrating T cells in urothelial bladder carcinoma (UBC) remains elusive. Here, single-cell RNA sequencing (scRNA-seq) were performed on total 30,905 T cells derived from peripheral blood, adjacent normal and tumor tissues from two UBC patients. We identified 18 distinct T cell subsets based on molecular profiles and functional properties. Specifically, exhausted T (T_Ex_) cells, exhausted NKT (NKT_Ex_) cells, Ki67^+^ T cells and B cell-like T (B-T) cells were exclusively enriched in UBC. Additionally, the gene signatures of T_Ex_, NKT_Ex_, Ki67^+^ T and B-T cells were significantly associated with poor survival in patients with BC and various tumor types. Finally, *IKZF3* and *TRGC2* are the potential biomarkers of T_Ex_ cells. Overall, our study demonstrated an exhausted context of T cells in UBC, which layed a theoretical foundation for the development of effective tumor immunotherapies.

## Introduction

Bladder cancer (BC) remains as one of the serious threats to public health recorded worldwide, and this disease has a higher prevalence in males in comparison to females^1,2^. Unfortunately, myriad BC therapeutic treatments included surgery, chemotherapy, and radiotherapy did not sustain a long-life expectancy or a decreased recurrence rate^3,4^.

According to histological classification, BC is divided into urothelial baldder carcinoma (UBC), squamous cell carcinoma, small cell carcinoma, adenocarcinoma, etc. BC usually occurs in the urethra epithelium, and more than 90% of BC patients are diagnosed with UBC. Squamous cell carcinoma and adenocarcinoma account for about 5% and 2%, respectively^5,6^. The cancer only affecting the urothelium or lamina propria is called non-muscle-invasive bladder cancer (NMIBC), while the cancer invading the original muscle is defined as muscle-invasive bladder cancer (MIBC)^7^. The treatment of UBC is mainly surgical treatment, with chemotherapy and radiotherapy playing an auxiliary role. The standard treatment for patients with NMIBC is bladder preservation, transurethral resection, and postoperative adjuvant chemotherapy. The standard treatment for patients with MIBC is neoadjuvant chemotherapy and radical cystectomy^6^. However, for patients with advanced and metastatic UBC, the choice of treatment methods is very limited.

Immunotherapy had drawn tremendous attention from UBC treatment recently. In the clinic, *Mycobacterium bovis bacillus Calmette-Guerin* (BCG) vaccine has achieved great success in UBC immunotherapy^8^ and the administration of five immune checkpoint agents had been approved by the Food and Drug Administration (FDA)^4^. Albeit the durable responses from immune checkpoint blockade, the clinical outcomes were not uniform and some undesirable side effects were experienced by the patients^9^. With the progressive advance in technology, single-cell RNA sequencing (scRNA-seq) analysis allows the detailed characterization of immune signatures of tumor microenvironment^10–12^. Compelling evidence demonstrated that this powerful tool initiated the deep interrogation of phenotype and functional heterogeneity among tumor resident immune cell populations in non-small-cell lung cancer^13^, breast cancer^14^ and liver cancer^15^. Elucidation of the immune repertoires allows the discovery of signatures that cause the failure in immunotherapy^11^. Therefore, this integrated analysis of immune signatures via scRNA-seq is like a beacon of guiding the procedures of immunotherapy.

Thus, the deep investigation of sophisticated immune regulation in the microenvironment of UBC provided cherishing information in amending the optimal immunotherapy for UBC patients^2,16^. However, the composition and function of T cells populations in UBC remained elusive. Here, scRNA-seq of the T cell populations in peripheral blood mononuclear cells (PBMC), adjacent normal and tumor tissues from two MIBC patients were performed. The exclusively enriched T cell clusters and their gene expression signatures in UBC provide the promising targets for the development of robust and effective immunotherapy strategies towards UBC.

## Materials and methods

### Human specimens

 Primary human UBC, normal bladder and peripheral blood samples of UBC patients were obtained from the Second Affiliated Hospital of Kunming Medical University (Kunming, China) with informed consent and approved by the Research Ethics Committee of the Second Affiliated Hospital of Kunming Medical University. All experiments were performed in accordance with the institutional guidelines. Based on Supplementary Table 1, The patients did not suffer from autoimmune diseases and cancers other than UBC. Besides, they did not experience neoadjuvant chemotherapies or other anti-tumor therapies.

### Sample/single-cell preparation

The PBMC were isolated from the patients using lymphocyte separation medium (Mediatech, USA) according to the manufacturer’s protocol. Briefly, the blood samples were collected from the patients who were starved overnight in a purple blood tube containing EDTA anticoagulant (BD, USA). After the dilution with PBS, 6 mL of blood samples were layered carefully on top of the lymphocyte separation medium. After the centrifugation at 500*g* for 30 min at RT, the interlayers, between the blood plasma and lymphocyte separation medium, which were the PBMCs, were collected and added with 2–3 mL of red blood cell lysis buffer (Solarbio, China) to lyse the red blood cells at 4 °C for 8 min. After the centrifugation at 400*g* for 10 min at 4 °C, the cells were carefully transferred to a new tube and washed twice with 10 mL RPMI-1640 culture medium (Corning, USA). After the centrifugation, the cells sediment were collected and resuspended in the culture medium.

The UBC tissue and adjacent normal bladder tissues (NBT) were cut into small pieces using a sterilized cutter. Subsequently, both tissues were digested with 2 mL of collagenase IV (Yuanye Biotechnology, China) in a CO_2_ incubator for 2 h at 37 °C until homogenized. The cells were filtered with 40 µm cell strainer (Falcon, USA) and followed by the centrifugation at 1000–2000 rpm for 10 min. After discarding the supernatant, the cells were incubated in 4 °C for 8 min with red blood cell lysis buffer to lyse the red blood cells. Then, the cells were washed twice and resuspended in the culture medium.

### Magnetic-activated cell sorting of CD3 cells

The magnetic-activated cell sorting (MACS) of CD3^+^ T cells was performed using pre-cooled solutions. Firstly, the cells were resuspended in 80 µL PBS buffer and followed by the addition of 20 µL CD3 Microbeads, human (Miltenyi Biotec, Germany) for every 1 × 10^7^ cells. After incubation at 4 °C for 15 min, the cells were diluted with 1–2 mL of pre-cooled buffer and followed by the centrifugation at 300 g for 10 min. After resuspension in 500 µL pre-cooled buffer, the cells were transferred to the top of the MS column (Miltenyi Biotec, Germany). The flow-through was collected which contained the CD3^−^ cells. The MS columns were washed 3 times with 500 µL pre-cooled buffer to complete flow-through of CD3^-^ cells. After removing MS columns from MACS separator, the remaining flow throughs were collected to a new centrifuge tube, which was the CD3^+^ cells. The cell number was counted using 0.4% trypan blue (Mediatech, USA) and maintained at a concentration of 7 × 10^5^ to 1.2 × 10^6^ cells/mL. The cells were retained on ice before proceeding to further experiments.

### Immunofluorescent staining/flow cytometry of T cell determination

After sorting out the CD3^+^ cells, the determination of T cell populations was performed via flow cytometry detection. Firstly, 1 × 10^6^ CD3^+^ cells were stained with 10 µL of FITC Anti-Human CD3 (OKT-3) Monoclonal Antibody, PE/Cy5 Anti-Human CD4 (OKT-4) Monoclonal Antibody and APC Anti-Human CD8 (HIT8a) Monoclonal Antibody (SunGene Biotech, China) together and incubated for 30 min at 4–8 °C in dark condition. After centrifugation and washing the cells with 500 µL PBS, the stained cells were resuspended with 500 µL of RPMI-1640 medium. The flow cytometry analysis of T cell populations was performed using FACSCalibur (BD, USA) and T cell populations were analyzed using FlowJo software.

### ScRNA-seq

The sorted cells were counted and assessed for the viability with 0.4% trypan blue using LUNA-II™ automated cell counter (Logosbio, Korea). The cells were then maintained at a concentration of 7 × 10^5^ to 1.2 × 10^6^ cells/mL with final viability of > 80% for the subsequent sequencing. The cell suspension was loaded into Chromium microfluidic chips and barcoded with a 10 × Chromium Controller (10 × Genomics). The RNA from barcoded cells was reverse-transcribed. Subsequently, single-cell library preparation was constructed using the reagents from a Chromium Single Cell reagent kit (10 × Genomics) according to the manufacturer’s protocols. Sequencing was performed with Illumina (Logosbio, Korea) according to the manufacturer’s protocols.

### ScRNA-seq data processing

The sequenced data from Illumina sequencer (Logosbio, Korea) were first processed to filter out the low-quality reads via trimmomatic software which can be summarized below: (1) average quality per base drops below 10; (2) “N” bases quality below 3; (3) containing adaptor sequence; (4) droping reads below the 26 bases long and (5) not forming paired reads. The remaining reads passed the filtering criteria were counted as clean reads and converted to Fastq format from BCL format for the subsequent analyses. Provided by 10 × Genomics, the Cell Ranger software pipeline (Version 3.0.2) was used to demultiplex cellular barcodes, align the reads to the reference genome and transcriptome using STAR aligner, thereby building up a matrix of the gene expressed in each cell. The unique molecular identifier (UMI) count matrix was processed using the R package Seurat (Version 2.3.0).

Since scRNA-seq implicated noise, quality-control (QC) assessments were performed after the basic data processing following the mentioned criteria. The number of genes detected in each cell must be within the range of 200–4000 based on the sample and the least number of cells needed for the gene detection was three. Besides, the ratio of UMI for mitochondrial gene number (percent.mito) and UMI for haemoglobin gene number (percent.HB) were not exceeding 0.3 and 0.05, respectively. The information on QC criteria for further processing as detailed in Supplementary Table 2. After applying these QC criteria, a total of 30,905 cells were included for further downstream analyses.

### Dimensionality reduction and clustering

Based on the filtered cell and gene matrices, Seurat (Version 2.3.0) in R package was applied to normalize the gene expression matrices. The gene expression level was divided by the total gene expression in the cells, which was then converted to relative abundance and multiplied with the normalization factor (default is 10,000) before undergoing log transformation. Then, Seurat package was used to establish the mean–variance relationship of the normalized counts of each gene in every cell. Before dimensionality reduction and clustering, QC control was conducted to remove the source of variation such as background noise, cell cycle and batch effects. Clustering analysis was performed to identify the cell subsets. In dimensionality reduction, principal component analysis (PCA) using Seurat package was performed to identify the significant principal component for the further clustering. A graph-based clustering algorithm using k-nearest neighbors (KNN) algorithms integrated with louvain community detection was conducted for the clustering of cell subsets. The t-SNE maps were generated to visualize the cluster of cells in two dimensions.

### Differential gene expression analysis and gene annotation

Seurat (Version 2.3.0) was used to perform differential gene expression analysis between different cell clusters. Using the Wilcoxon Rank Sum Test, the differentially expressed genes (DEGs) were determined. The highly expressed DEGs in each cell clusters were presented in a heat map.

### DEGs enrichment analysis and protein–protein interaction analysis

Based on the t-SNE map, the cell cluster which has the highly expressed DEGs were selected for gene annotation, gene-set enrichment analysis and protein–protein interaction analysis. These analyses were performed using Metascape (https://metascape.org/) to produce a high-quality graphical presentation. The enrichment analysis was based on hypergeometric distribution and p-adjusted (padj) which was less than 0.01 signified significant enrichment. The overlapped gene regions were visualized using Circos software (http://circos.ca/).

### Analysis of the correlation between stage, grade, and neutrophil–lymphocyte ratio (NLR) with T cell subpopulations

In order to better understand the heterogeneity of T cell subsets in tumor microenvironment and validate above results in more samples, immunofluorescence staining was carried out and examined by microscope in additional 30–50 UBC samples with different stages (Supplementary Table 1). According to previous reports, the UBC case with NLR ≥ 2.5 is defined as high and that NLR < 2.5 is defined as low^17^. Graphpad Prism 8.0 was applied to analyze the relationship between T cell subpopulations and stage, grade, and NLR in UBC patients.

### Prognostic and survival analysis

The overall survival (OS) and disease-free survival (DFS) of the gene signatures of cell cluster were analyzed by GEPIA2 (http://gepia2.cancer-pku.cn/#index). The prognostic value of the respective gene signatures in different cell clusters was evaluated using Kaplan–Meier curves in GEPIA2.

### Statistical analysis

The Student t test was used to compare the mean values of two groups. In the gene expression and survival analysis, the average of gene expression was first calculated. UBC samples expressing signature genes of exhausted T (T_Ex_), exhausted NKT (NKT_Ex_), KI67^+^ T and B cell-like T (B-T) cells were defined as the high signatures group and the remaining samples as the low signatures group. The OS of each group was calculated by a Kaplan–Meier analysis, and the difference between those two groups was examined using the log rank test. A value of *P* less than 0.05 was regarded as statistically significant (*P < 0.05, **P < 0.01 and ***P < 0.001).

## Results

### Single-cell isolation and sequencing of tumor-infiltrating T cells in human UBC

**Figure 1:**
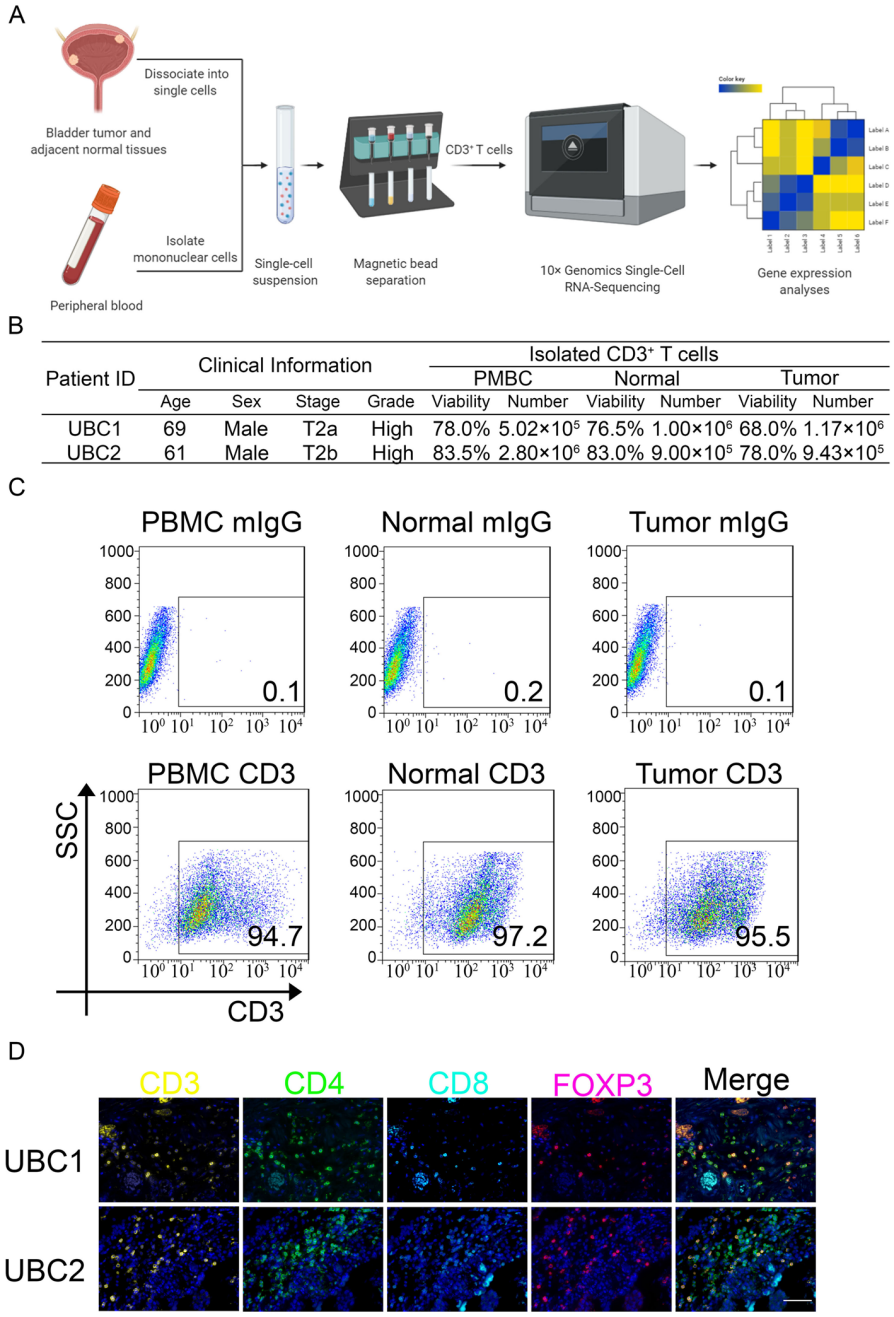
Dissociation of tumor-infiltrating T Cells from UBC patients (**A**) Overview of the study design. (**B**) The UBC patient information, and the viability and numbers of T cells isolated. (**C**) Confirmation of the percentage of CD3 positive T cells of magnetic beads isolated single-cells by FACS Calibur. (**D**) Representative immunofluorescent staining with anti-CD3, CD4, CD8, and FOXP3 antibodies in UBC. Bar = 125 μm.

To better decipher the heterogeneity of tumor-infiltrating T cells in UBC in single-cell level, the CD3^+^ T cells were isolated from PBMC, adjacent normal and tumor tissues from two MIBC patients (UBC1 and UBC2) via magnetic bead separation method (Fig. 1A). The qualified concentration, viability and purity of isolated T cells ensured single cell library preparation (Fig. 1B,C). Additionally, multicolor immunofluorescent staining of T cell population were performed to determine the T cell subsets in UBC by the antibodies targeting CD3, CD4, CD8 and FOXP3 (Fig. 1D). Taken together, CD3^+^ T cells were successfully isolated from PBMC, adjacent normal and tumor tissues from UBC patients.

### Landscape of tumor-infiltrating T cells in UBC1

The acquired qualified CD3^+^ T cells were sequentially analyzed by scRNA-seq (Fig. 1A). The single-cell sequencing data and filteration parameters were summarized in Supplementary Fig. 1 and Supplementary Tables 2, 3. After filteration, a total of 30,905 T cells were selected for T cell subsets analysis according to gene expression signature (Supplementary Figs. 2, 3).

**Figure 2:**
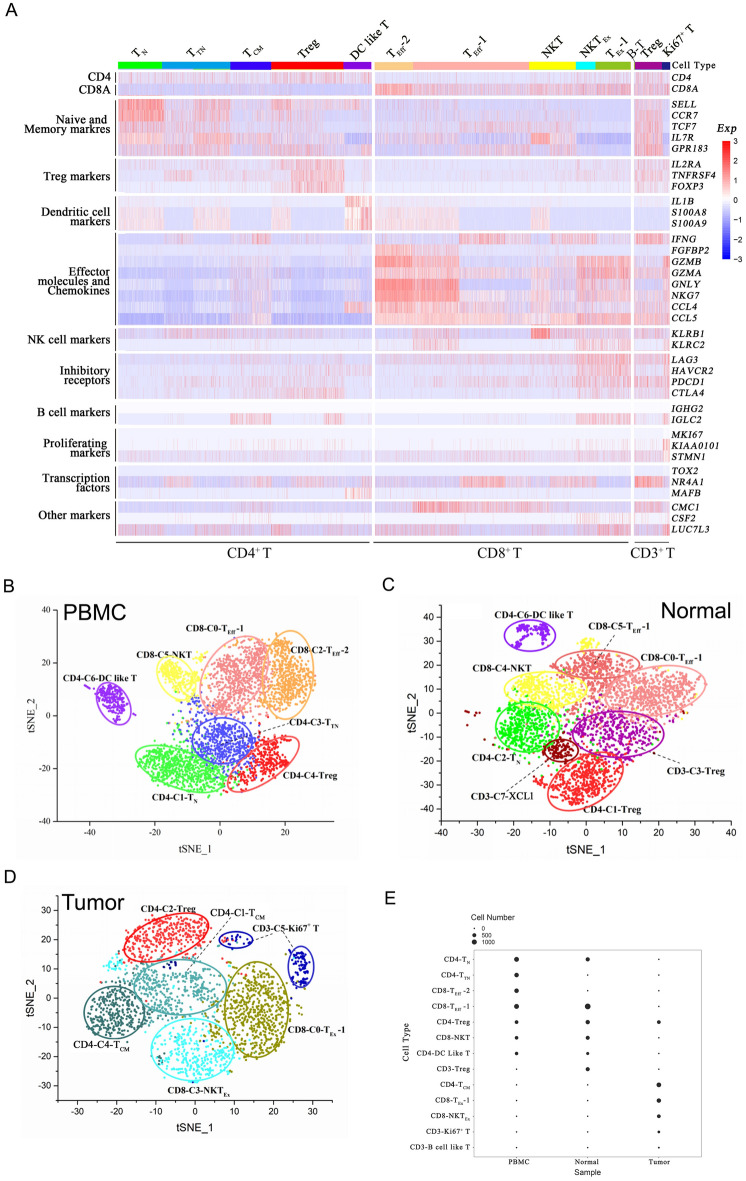
Single-cell profiling of tumor-infiltrating T cells in UBC1 (**A**) Heat map from single-cell analysis via expression recovery (SAVER) imputed data with cells grouped into clusters (indicated by colored bars at the top). The top ten genes differentially expressed for each cluster are shown on the y axis, and key genes are also shown for each cluster. (**B**) The t-SNE projection of T cells of PBMC from UBC1, showing the formation of seven main clusters shown in different colors. (**C**) The t-SNE projection of T cells of NBT from UBC1, showing the formation of eight main clusters shown in different colors. (**D**) The t-SNE projection of T cells of UBC tissue from UBC1, showing the formation of six main clusters shown in different colors. (**E**) The frequencies of each cluster within the PBMC, NBT, and UBC tissue samples are depicted by balloon plots.

A total of 10,895 T cells were identified in PBMC, NBT, and UBC tissue from UBC1. Through clustering analysis, a graph based clustering algorithm is used, which constructs a KNN graph through euclidean distance. By default, louvain algorithm is used to group cells and optimize modules. Based on the clustering results, 13 clusters of T cell populations were displayed using the t-SNE dimensionality reduction algorithm (Fig. 2A). Based on this, the expression of *CD3D*, *CD4*, *CD8A*, and *FOXP3* in each cluster was further analyzed (Supplementary Fig. 4A–C). Seven, eight and six T subsets were identified in PBMC, NBT and UBC sample, respectively (Fig. 2B–D). The composition of the T cell population in PBMC was similar to that of NBT (Fig. 2E). Notably, cluster 0 and cluster 2 from PBMC were effector T cells and named as CD8-C0-T_Eff_-1 and CD8-C2-T_Eff_-2, respectively (Fig. 2B). Similarly, cluster 0 and cluster 5 from NBT were effector T cells and named as CD8-C0-T_Eff_-1 and CD8-C5-T_Eff_-1, respectively (Fig. 2C). They highly expressed various effector molecules-related genes, such as *CCL4*, *GZMA*, *IFNG*, *NKG7* and *KLRG1*^18^ (Fig. 2A, Supplementary Fig. 2A and Supplementary Tables 4–6).

In addition, cluster 1 from PBMC and cluster 2 from NBT were naïve T cells and named as CD4-C1-T_N_ and CD4-C2-T_N_, respectively, which highly expressed various naïve T cell markers, such as *CCR7* and *SELL*^19^, not effector molecules (Fig. 2A–C, Supplementary Fig. 2A and Supplementary Tables 4–6). Furthermore, cluster 3 from PBMC was transitional naïve T cells, named as CD4-C3-T_TN_, which highly expressed *GPR171*, *GPR183*, *LTB* and *RGS1*, not effector molecules (Fig. 2A–B, Supplementary Fig. 2A and Supplementary Tables 4, 5). Compared with T_N_, the expression of *CCR7*, *LEF1*, *MYC*, *SELL* and *TCF7* decreased in T_TN_, which represented a transition state from T_N_ cells into T_CM_ cells.

Moreover, cluster 4 from PBMC, cluster 1 from NBT and cluster 2 from UBC were Treg cells and named as CD4-C4-Treg, CD4-C1-Treg and CD4-C2-Treg, respectively (Fig. 2B–D), which highly expressed Treg cell markers, such as *CTLA4*, *FOXP3*, *IL2RA* and *TNFRSF4*^15^ (Fig. 2A, Supplementary Fig. 2A–C, Supplementary Tables 4–6). Surprisingly, cluster 3 from NBT, named as CD3-C3-Treg, not only expressed Treg cell markers (*CTLA4*, *FOXP3*, *IL2RA* and *TNFRSF4*), but also T cell exhaustion-related genes, such as *LAG3*, *PDCD1* and *NR4A1* (Fig. 2A,C).

Furthermore, cluster 5 from PBMC and cluster 4 from NBT were NKT cells, named as CD8-C5-NKT cells and CD8-C4-NKT cells, respectively (Fig. 2B,C), which highly expressed NK cell markers, such as *KLRB1*, *CD69*^20^ and granzyme encoding genes (*GZMA*, *GZMB*, *GZMH* or *GZMK*)^21^ (Fig. 2A–C, Supplementary Fig. 2A–B, Supplementary Tables 4–6). Interestingly, cluster 6 from PBMC and cluster 6 from NBT were highly expressed DC markers, such as *IL1β*, *S100A8* and *S100A9*^22^, which were named as CD4-C6-DC like T cells (Fig. 2A–C and Supplementary Fig. 2A–B, Supplementary Tables 4–6). In NBT, a special cluster, named as CD3-C7-XCL1, was CD4^−^CD8^−^ and highly expressed a chemokine superfamily encoding gene *XCL1* (Fig. 2C and Supplementary Fig. 2B), representing the developmental stage of pro-T cells.

The composition of the T cell populations in UBC was remarkably different compared to those of PMBC or NBT (Fig. 2D–E). Cluster 0 (CD8-C0-T_Ex_-1) and cluster 3 (CD8-C3-NKT_Ex_) were dominate in UBC and highly expressed exhaustion-related genes *LAG3*, *HAVCR2*, *PDCD1* and *CTLA4* (Fig. 2A and Supplementary Table 7), suggesting the exhausted phenotype in T cells in UBC microenvironment. Specifically, CD8-C3-NKT_Ex_ highly expressed NK cell surface receptor coding genes *KLRB1*, *KLRC2* and *KLRD1.* Interestingly, cluster 1 (CD4-C1-T_CM_) and cluster 4 (CD4-C4-T_CM_) from UBC were T_CM_ cells, which highly expressed T_CM_ cell markers, such as *IL7R* and *CCR7*^23^ (Fig. 2A,D, Supplementary Fig. 2C, Supplementary Table 7). Intriguingly, a small subset of CD4-C1-T_CM_ in UBC, representing 2.2% of the unique cell clusters, highly expressed immunoglobulin-related genes, such as *IGHA1*, *IGHG1-4 and IGLC2-3* (Supplementary Table 4), which were responsible for antibody synthesis of B cells. More importantly, cluster 5 (CD3-C5-Ki67^+^ T) was distinctively found in UBC and divided into two groups (Fig. 2D,E). This cell cluster expressed high levels of proliferation factors *MKi67*, *KIAA0101* and *STMN1* (Supplementary Table 7), suggesting the rapid proliferation state. In short, there is a high similarity between the T cell population in PBMC and NBT of UBC1, and T_Ex_-1, NKT_Ex_, B-T and Ki67^+^ T cells were exclusively enriched in UBC, which potentialy participated in the formation of the immunosuppressive microenvironment of UBC.

### Profile of tumor-infiltrating T cells in UBC2

**Figure 3:**
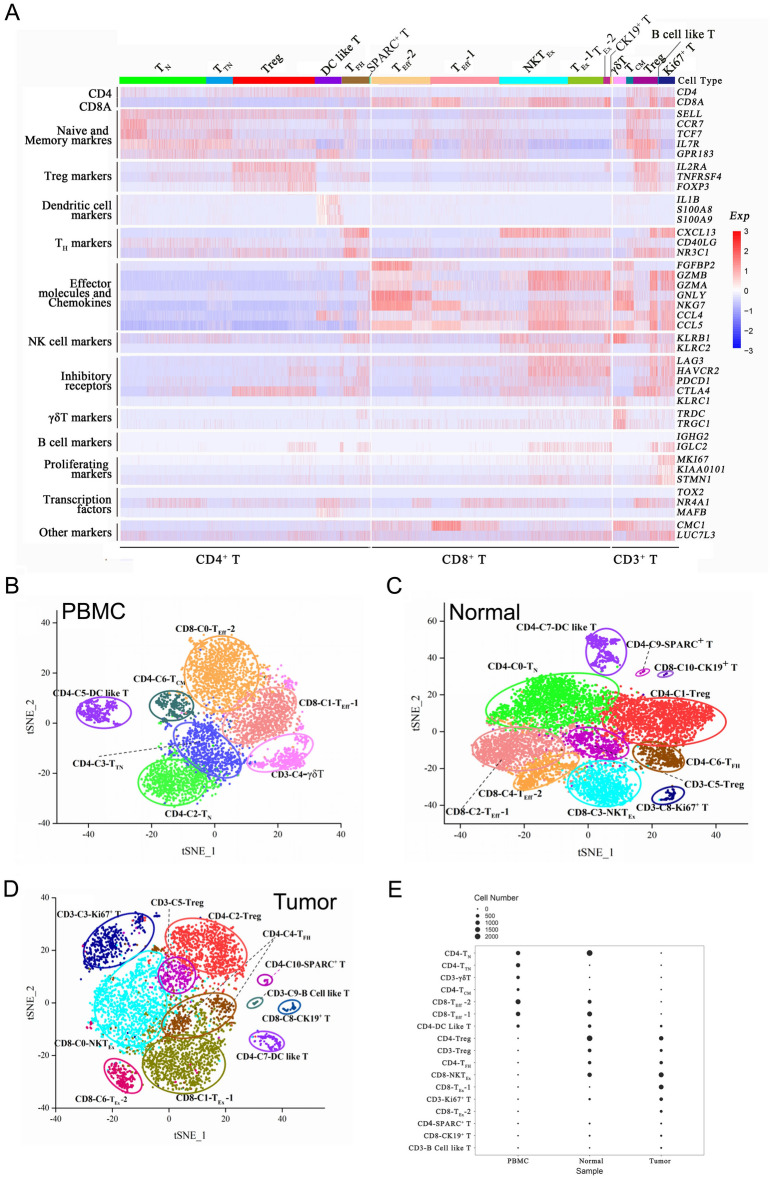
Single-cell analysis of tumor-infiltrating infiltrating T cells in UBC2 (**A**) Heat map from SAVER imputed data with cells grouped into clusters (indicated by colored bars at the top). The top ten genes differentially expressed for each cluster are shown on the y axis, and key genes are also shown for each cluster. (**B**) The t-SNE projection of T cells of PBMC from UBC2, showing the formation of seven main clusters shown in different colors. (**C**) The t-SNE projection of T cells of NBT from UBC2, showing the formation of 11 main clusters shown in different colors. (**D**) The t-SNE projection of T cells of UBC tissue from UBC2, showing the formation of 11 main clusters shown in different colors. (**E**) The frequencies of each phenograph cluster within the PBMC, NBT, and UBC tissue samples are depicted by balloon plots.

A total of 20,010 T cells were characterized in PBMC, NBT and UBC from UBC2. Through clustering analysis, a graph based clustering algorithm is used, which constructs a KNN graph through euclidean distance. By default, louvain algorithm is used to group cells and optimize modules. Based on the clustering results, 17 clusters of T cell populations were displayed using the t-SNE dimensionality reduction algorithm (Fig. 3A and Supplementary Fig. 3). Based on this, the expression of *CD3D*, *CD4*, *CD8A*, and *FOXP3* of each cluster were analyzed (Supplementary Fig. 4D,F).

There were seven clusters of T cell populations determined in PBMC (Fig. 3B), which were similar to those of PBMC from UBC1. T_Eff_ (CD8-C0-T_Eff_-1, CD8-C2-T_Eff_-2), T_N_ (CD4-C1-T_N_), T_TN_ (CD4-C3-T_TN_) and DC like T cells (CD4-C5-DC like T) were also identified (Supplementary Table 8). Notably, cluster 4 from PBMC was γδT cells and named as CD3-C4-γδT, which highly expressed γδT cells-related genes, such as *TRDC* and *TRGC1*^24^, and effector molecules and chemokines encoding genes *GNLY*, *NKG7* and *CCL5*, suggesting the potential of cytotoxicity induction (Fig. 3A and Supplementary Table 4). Furthermore, T_CM_ (CD4-C6-T_CM_) had high expression of *IL7R*, *GPR183, GNLY* and *NKG7*, whose expression was similar to that of T_CM_ from UBC1 (Supplementary Table 8).

There were 11 groups of T cell clusters found in the NBT (Fig. 3C) and T_N_ (CD4-C0-T_N_), Treg (CD4-C1-Treg, CD3-C5-Treg), T_Eff_ (CD8-C2-T_Eff_-1, CD8-C4-T_Eff_-2) and DC like T cells (CD4-C7-DC like T) were also identified (Supplementary Table 9). Interestingly, the CD8-C3-NKT_Ex_ cluster expressed high levels of exhaustion-related genes *LAG3*, *HAVCR2* and *CTLA4,* and NK cell surface receptor conding genes *KLRB1*, *KLRC2* and *KLRD1* (Fig. 3C and Supplementary Table 9)*.* Moreover, the CD3-C8-Ki67^+^ T cluster expressed high levels of proliferation factors *MKi67*, *KIAA0101* and *STMN1*^14^, suggesting the rapid proliferation state (Fig. 3C and Supplementary Table 9).

Three additional T cell types, namely CD4-C6-T_FH_, CD4-C9-SPARC^+^ T and CD8-C10-CK19^+^ T cells, were identified (Fig. 3C and Supplementary Table 9). Specifically, T_FH_ highly expressed helper T cell-related genes *CXCL13*, *CD40LG* and *NR3C1*^25^*.* SPARC^+^ T cells highly expressed the genes responsible for extracellular matrix formation, *SPARC*, *VEGFA*, *A2M* and *COL4A1*^26^, thus implicating an important role in cancer development and metastasis (Supplementary Table 9). Meanwhile, CK19^+^ T cells population expressed high levels of keratin family-related genes *KRT19*, *KRT17* and *KRT7*, which was newly discovered in UBC.

There were 11 groups of T cell clusters found in the UBC (Fig. 3D). Similar to the T cell distribution of NBT from UBC2, NKT_Ex_ (CD8-C0-NKT_Ex_), Treg (CD4-C2-Treg, CD3-C5-Treg), Ki67^+^ T (CD3-C3-Ki67^+^ T), T_FH_ (CD4-C4-T_FH_), DC like T cells (CD4-C7-DC like T), CK19^+^ T (CD8-C8-CK19^+^ T) and SPARC^+^ T (CD4-C10-SPARC^+^ T) were also identified. T_Ex_ (CD8-C1-T_Ex_-1, CD8-C6-T_Ex_-2) and B-T cells (CD3-C9-B cell like T) were exclusively enriched in UBC from UBC2 (Fig. 3D). The composition of the T cell populations in UBC was remarkably different compared to those of PBMC or NBT (Fig. 3E).

### Gene expression signature of exhausted CD8^+^ T cells

**Figure 4:**
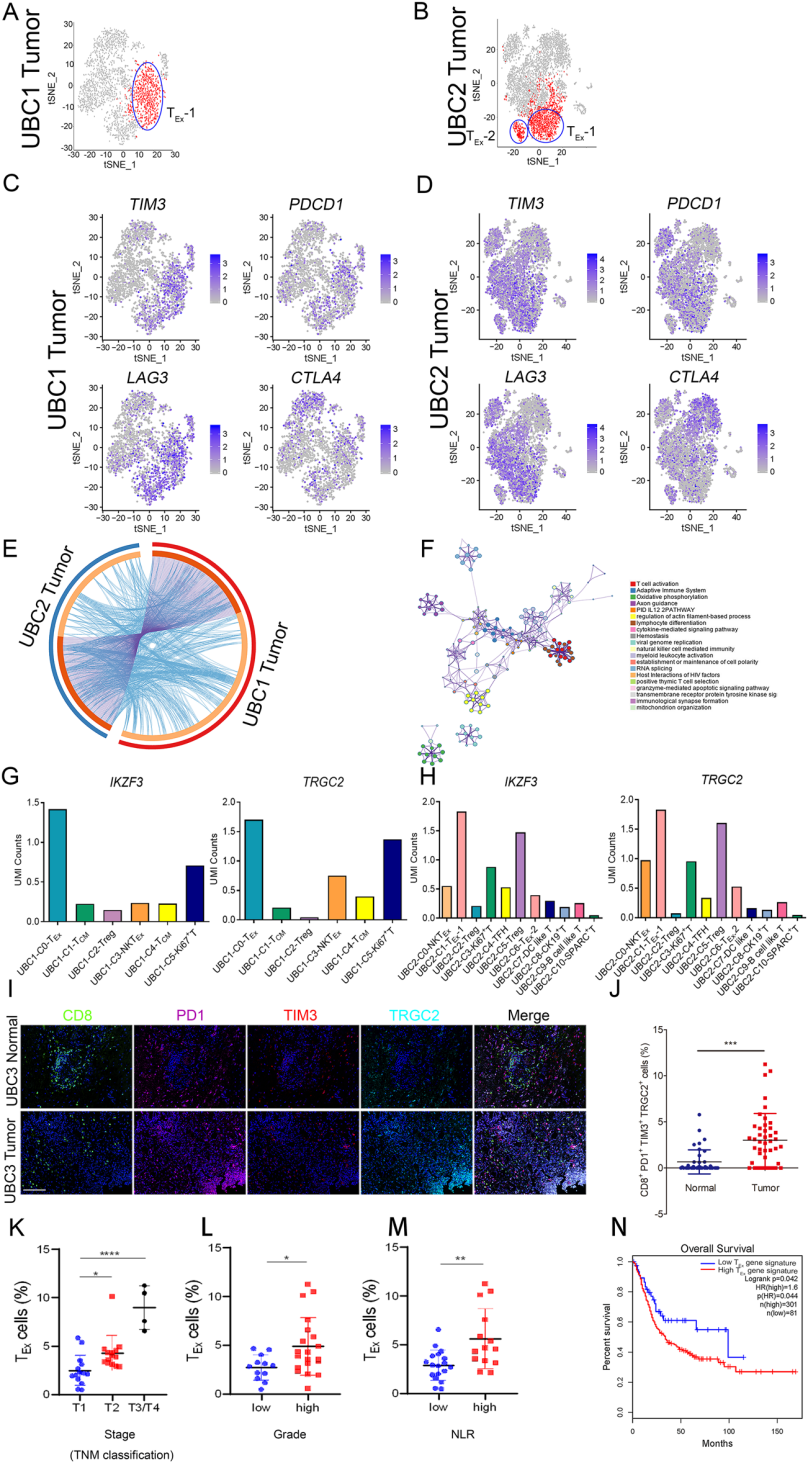
Characterization of exhausted CD8^+^ T Cells and its clinical implication in UBC (**A**, **B**) The t-SNE projection of exhausted CD8^+^ T cells (red) in tumor tissues of UBC1 and UBC2. (**C**, **D**) Expression levels of *TIM3*, *PDCD1*, *LAG3* and *CTLA4*, across 7939 (UBC1 2446 and UBC2 5493) single T cells illustrated in t-SNE plots. (**E**) The gene expression status between tumor tissues of UBC1 and UBC2 were visualized in a circular layout via the Circos analysis tool. The red line of the outer circle manifests UBC1, and the blue one stands for UBC2. The orange line of the inner circle manifests the common genes shared by both UBC1 and UBC2. The light orange line in the circle indicates the functional correlation between genes. (**F**) Pathway enrichment of 487 (UBC1 273, UBC2 214) signature genes in T_Ex_. (**G**) UMI counts of *IKZF3* and *TRGC2* in C0-T_Ex_-1, C1-T_CM_, C2-Treg, C3-NKT_Ex_ and C4-T_CM_ clusters in UBC1. (**H**) UMI counts of *IKZF3* and *TRGC2* in C0-NKT_Ex_, C1-T_Ex_-1, C2-Treg, C6-T_Ex_-2, C7-DC like T, C8-CK19^+^ T, C9-B cell like T and C10-SPARC^+^ T clusters in UBC2. (**I**) Representative immunofluorescent staining with anti-CD8, PD1, TIM3 and TRGC2 antibodies in NBT and UBC tissues. Bar = 125 μm. (**J**) The parentage of CD8^+^PD1^+^TIM3^+^TRGC2^+^ T_Ex_ in NBT and UBC tissues. (**K**) Correlation analysis between staging and T_Ex_ cells in UBC patients. (**L**) Correlation analysis between grading and T_Ex_ cells in UBC patients. (**M**) Correlation analysis between NLR and T_Ex_ cells in UBC patients. (**N**) Kaplan–Meier curves comparing the OS between UBC patients expressing high or low levels of gene signature in T_Ex_, log-rank test. n, patient number. Data are presented as mean ± SD. *P < 0.05, **P < 0.01.

Based on the global characterization of tumor-infiltrating T cells from two MIBC patients, T_Ex_ cells dominated the microenvironment of UBC (Fig. 4A,B). Specifically, 273 and 201 differentially expressed genes were identified in T_Ex_ cells from UBC1 and UBC2, respectively (Supplementary Table 11). Obviously, T_Ex_ cells expressed high levels of immune checkpoints genes *TIM3*, *PDCD1*, *LAG3* and *CTLA4* (Fig. 4C,D), and the majority of the differentially expressed genes were overlapped between T_Ex_ cells from UBC1 and UBC2 (Fig. 4E). By gene annotation (GO) and pathway enrichment analysis, the differential expression genes of T_Ex_ cells significantly enriched in T cell activation, adaptive immune system and oxidative phosphorylation, etc. (Fig. 4F and Supplementary Fig. 5). Furthermore, *IKZF3* and *TRGC2* are novel biomarkers of T_Ex_ (Fig. 4G,H) and CD8^+^PD1^+^TIM3^+^TRGC2^+^ T_Ex_ significantly enriched in UBC (3.47%) compared to NBT (0.66%) (Fig. 4I,J). Specifically, the proportion of T_Ex_ cells enhanced with the increase of the stage (*P* < 0.001) (Fig. 4K). Similarly, UBC patients with high grade (P < 0.05) and NLR (*P* < 0.01) had a increased proportion of T_Ex_ (Fig. 4L,M).

Survival analyses via GEPIA2 database indicated that UBC patients acquiring high expression of T_Ex_ signature genes (Supplementary Table 12) had a decreased OS as compared to those of UBC patients acquiring low expression of these signature genes (Fig. 4N). Additionally, lung adenocarcinoma (LUAD), lung squamous cell carcinoma (LUSC), liver hepatocellular carcinoma (LIHC), brain lower grade glioma (LGG) and adrenocortical carcinoma (ACC) patients acquiring high expression of T_Ex_ signature genes also had a decreased OS and DFS as compared to those of tumor patients acquiring low expression of these signature genes (Supplementary Fig. 6). Taken together, T_Ex_ was exclusively identified in UBC, which highly expressed *IKZF3* and *TRGC2*. The gene expression signature of T_Ex_ was associated with poor prognosis of patients with UBC and multiple other tumor types.

### Gene expression profile of NKT_Ex_ cell population

**Figure 5:**
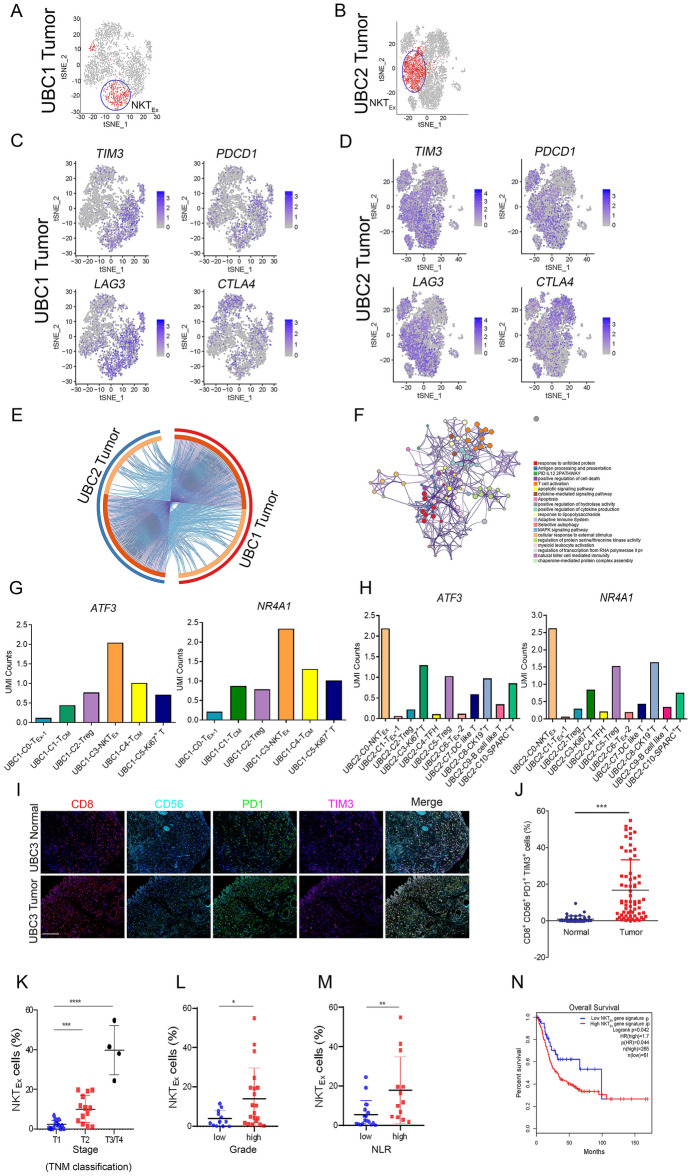
Characterization of NKT_Ex_ cells and its clinical implication in UBC (**A**, **B**) The t-SNE projection of NKT_Ex_ cells in tumor tissues of UBC1 and UBC2, showing in red. (**C**, **D**) Expression levels of *TIM3*, *PDCD1*, *LAG3* and *CTLA4*, across 7,939 (UBC1 2,446 and UBC2 5493) single T cells illustrated in t-SNE plots. (**E**) The gene expression status between tumor tissues of UBC1 and UBC2 were visualized in a circular layout via the Circos analysis tool. The red line of the outer circle manifests UBC1, and the blue one stands for UBC2. The orange line of the inner circle manifests the common genes shared by both UBC1 and UBC2. The light orange line in the circle indicates the functional correlation between genes. (**F**) Pathway enrichment of 315 (UBC1 164, UBC2 151) signature genes in NKT_Ex_. (**G**) UMI counts of *ATF3* and *NR4A1* in C0-T_Ex_, C1-T_CM_, C2-Treg, C3-NKT_Ex_ and C5-Ki67^+^ T clusters in UBC1. (**H**) UMI counts of *ATF3* and *NR4A1* in C0-NKT_Ex_, C1-T_Ex_-1, C2-Treg, C4-T_FH_, C6-T_Ex_-2, C7-DC like T and C9-B cell like T clusters in UBC2. (**I**) Representative immunofluorescent staining with anti-CD8, CD56, PD1 and TIM3 antibodies in NBT and UBC tissues. Bar = 125 μm. (**J**) The parentage of CD8^+^CD56^+^PD1^+^TIM3^+^ NKT_Ex_ in NBT and UBC tissues. (**K**) Correlation analysis between staging and NKT_Ex_ cells in UBC patients. (**L**) Correlation analysis between grading and NKT_Ex_ cells in UBC patients. (**M**) Correlation analysis between NLR and NKT_Ex_ cells in UBC patients. (**N**) Kaplan–Meier curves comparing the OS between UBC patients expressing high or low levels of gene signature in NKT_Ex_, log-rank test. n, patient number. Data are presented as mean ± SD. *P < 0.05, **P < 0.01.

Besides T_Ex_, NKT_Ex_ was another major subpopulation in UBC (Fig. 5A,B). Specifically, 165 and 152 differentially expressed genes were identified in NKT_Ex_ cells from UBC1 and UBC2, respectively (Supplementary Table 13). Similar to T_Ex_, NKT_Ex_ expressed high level of immune checkpoints such as *TIM3*, *PDCD1*, *LAG3* and *CTLA4* (Fig. 5C,D), and there was a high overlapping rate of specific genes among them (Fig. 5E). Moreover, the differential expression genes of NKT_Ex_ significantly enriched in the response to adaptive immune system, NK cell mediated immunity, T cell activation, etc. (Fig. 5F, Supplementary Fig. 7). Furthermore, *ATF3* and *NR4A1* were the important biomarkers in identification of NKT_Ex_ (Fig. 5G,H) and CD8^+^CD56^+^PD1^+^TIM3^+^ NKT_Ex_ significantly infiltrated into UBC (16.92%) rather than NBT (0.92%) (Fig. 5I,J). Compared with T1 stage, the proportion of NKT_Ex_ cells in T2 and T3/T4 stages was significantly increased (*P* < 0.001) (Fig. 5K). Similarly, UBC patients with higher grading (*P* < 0.05) and NLR (*P* < 0.01) had a higher proportion of NKT_Ex_ cells (Fig. 5L,M).

UBC patients acquiring high expression of NKT_Ex_ signature genes (Supplementary Table 14) had a decreased OS of UBC patients compared to those of UBC patients acquiring low expression of these signature genes (Fig. 5N). Additionally, LUAD, LUSC, LIHC, LGG and ACC patients acquiring high expression of NKT_Ex_ signature genes had a decreased OS and DFS as compared to those of tumor patients acquiring low expression of these signature genes. Taken together, NKT_Ex_ was significantly enriched in UBC (Supplementary Fig. 8), which highly expressed *ATF3* and *NR4A1*. The gene expression signature of NKT_Ex_ correlated with poor prognosis of patients with UBC and other tumor types.

### Ki67^+^ T cells are enriched in UBC

**Figure 6:**
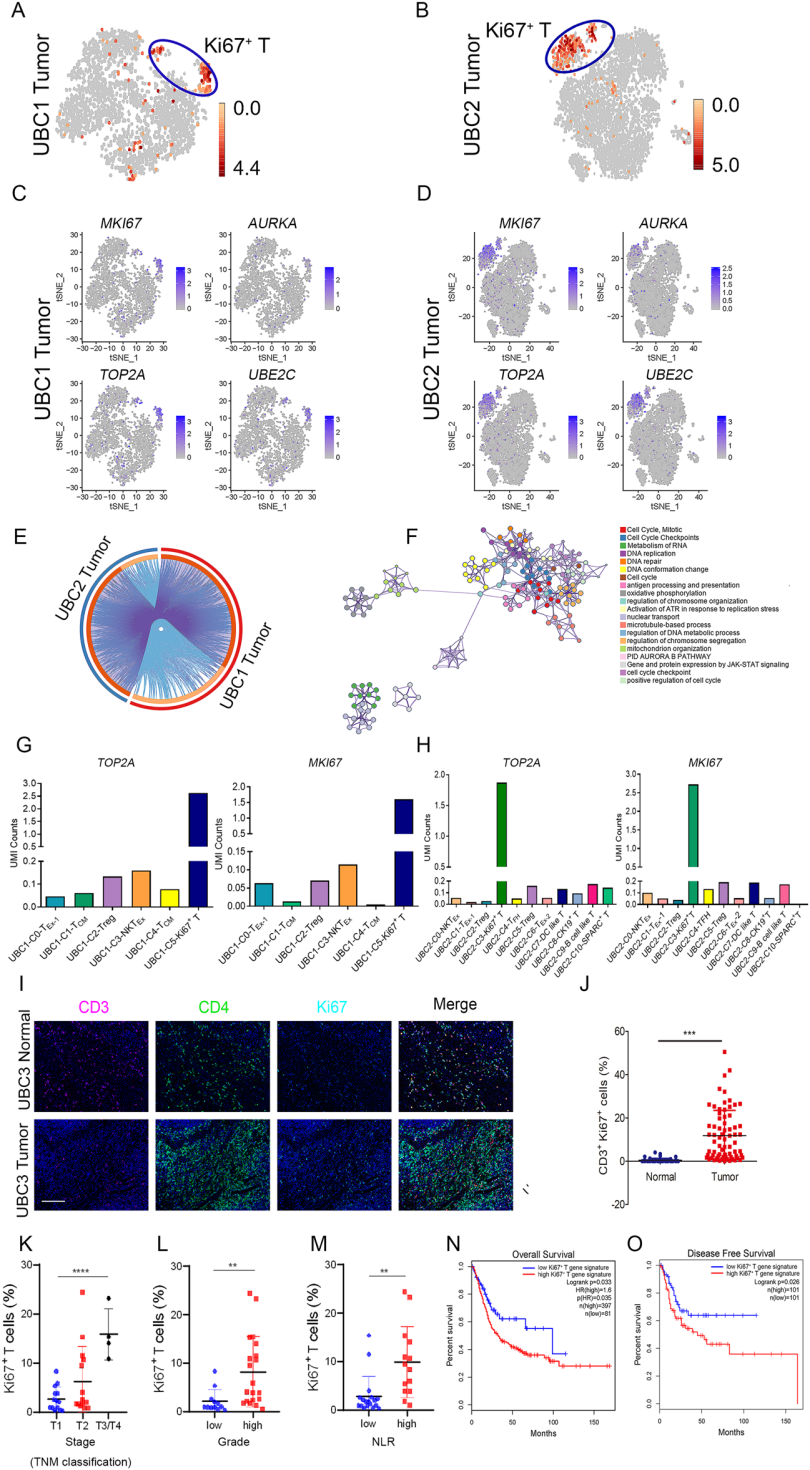
Characterization of Ki67^+^ T Cells and its clinical implication in UBC (**A**, **B**) The t-SNE projection of Ki67^+^ T cells in tumor tissues of UBC1 and UBC2, showing in red. (**C**, **D**) Expression levels of *MKI67*, *AURKA*, *TOP2A* and *UBE2C*, across 7939 (UBC1 2446 and UBC2 5493) single T cells illustrated in t-SNE plots. (**E**) The gene expression status between tumor tissues of UBC1 and UBC2 were visualized in a circular layout via the Circos analysis tool. The red line of the outer circle manifests UBC1, and the blue one stands for UBC2. The orange line of the inner circle manifests the common genes shared by both UBC1 and UBC2. The light orange line in the circle indicates the functional correlation between genes. (**F**) Pathway enrichment of 1247 (UBC1 736, UBC2 551) signature genes in Ki67^+^ T cells. (**G**) UMI counts of *KI67* and *TOP2A* in C0-T_Ex_, C1-T_CM_, C2-Treg, C3-NKT_Ex_, C4-T_CM_ and C5-Ki67^+^ T clusters in UBC1. (**H**) UMI counts of *KI67* and *TOP2A* in C0-NKT_Ex_, C1-T_Ex_-1, C2-Treg, C3-Ki67^+^ T, C4-T_FH_, C5-Treg, C6-T_Ex_-2, C7-DC like T, C8-CK19^+^ T, C9-B cell like T and C10-SPARC^+^ T clusters in UBC2. (**I**) Representative immunofluorescent staining with anti-CD3, CD4 and Ki67 antibodies in NBT and UBC tissues. Bar = 125 μm. (**J**) The parentage of CD3^+^ Ki67^+^Ki67^+^ T cells in NBT and UBC tissues. (**K**) Correlation analysis between staging and Ki67^+^ T cells in UBC patients. (**L**) Correlation analysis between grading and Ki67^+^ T cells in UBC patients. (**M**) Correlation analysis between NLR and Ki67^+^ T cells in UBC patients. (**N**, **O**) Kaplan–Meier curves comparing the OS and DFS between UBC patients expressing high or low levels of gene signature in Ki67^+^ T cells, log-rank test. n, patient number. Data are presented as mean ± SD. *P < 0.05, **P < 0.01.

The Ki67^+^ T cells were existed in both UBC1 and UBC2 (Fig. 6A,B). Specifically, 742 and 556 differentially expressed genes were identified in Ki67^+^ T cells from UBC1 and UBC2, respectively (Supplementary Table 15). Ki67^+^ T cells expressed high level of immune checkpoints such as *MKI67*, *AURKA*, *TOP2A* and *UBE2C* (Fig. 6C,D), and there was a high overlapping rate of specific genes among them (Fig. 6E). Moreover, the differential expression genes significantly enriched in the cell cycle, cell proliferation and DNA replication, suggesting an active proliferation status of Ki67^+^ T cell clusters (Fig. 6F, Supplementary Fig. 9). Furthermore, *MKI67* and *TOP2A* were the important biomarkers in identification of Ki67^+^ T cells (Fig. 6G,H). Additionally, immunofluorescence assay demonstrated that CD3^+^Ki67^+^ T cells significantly enriched in UBC (11.85%) compared to NBT (0.46%) (Fig. 6I,J).

Specifically, the proportion of Ki67^+^ T cells increased with the increment of stages (*P* < 0.001) (Fig. 6K). Similarly, as the grading (*P* < 0.01) and NLR (*P* < 0.01) of UBC patients increasd, the proportion of Ki67^+^ T cells augmented (Fig. 6L,M). The UBC patients with high expression of Ki67^+^ T signature genes (Supplementary Table 16) had a lower OS as well as DFS as compared to those of UBC patients acquiring low expression of these signature genes (Fig. 6N,O). Additionally, LUAD, LUSC, LIHC, LGG and ACC patients acquiring high expression of Ki67^+^ T signature genes had a decreased OS and DFS as compared to those of tumor patients acquiring low expression of these signature genes (Supplementary Fig. 10). Hence, Ki67^+^ T cells was remarkably enriched in UBC, which highly expressed *MKI67*, *AURKA*, *TOP2A* and *UBE2C*. The gene expression signature of Ki67^+^ T cells correlated with poor prognosis of patients with UBC and other tumor types.

### Gene expression landscape of B-T cells

**Figure 7:**
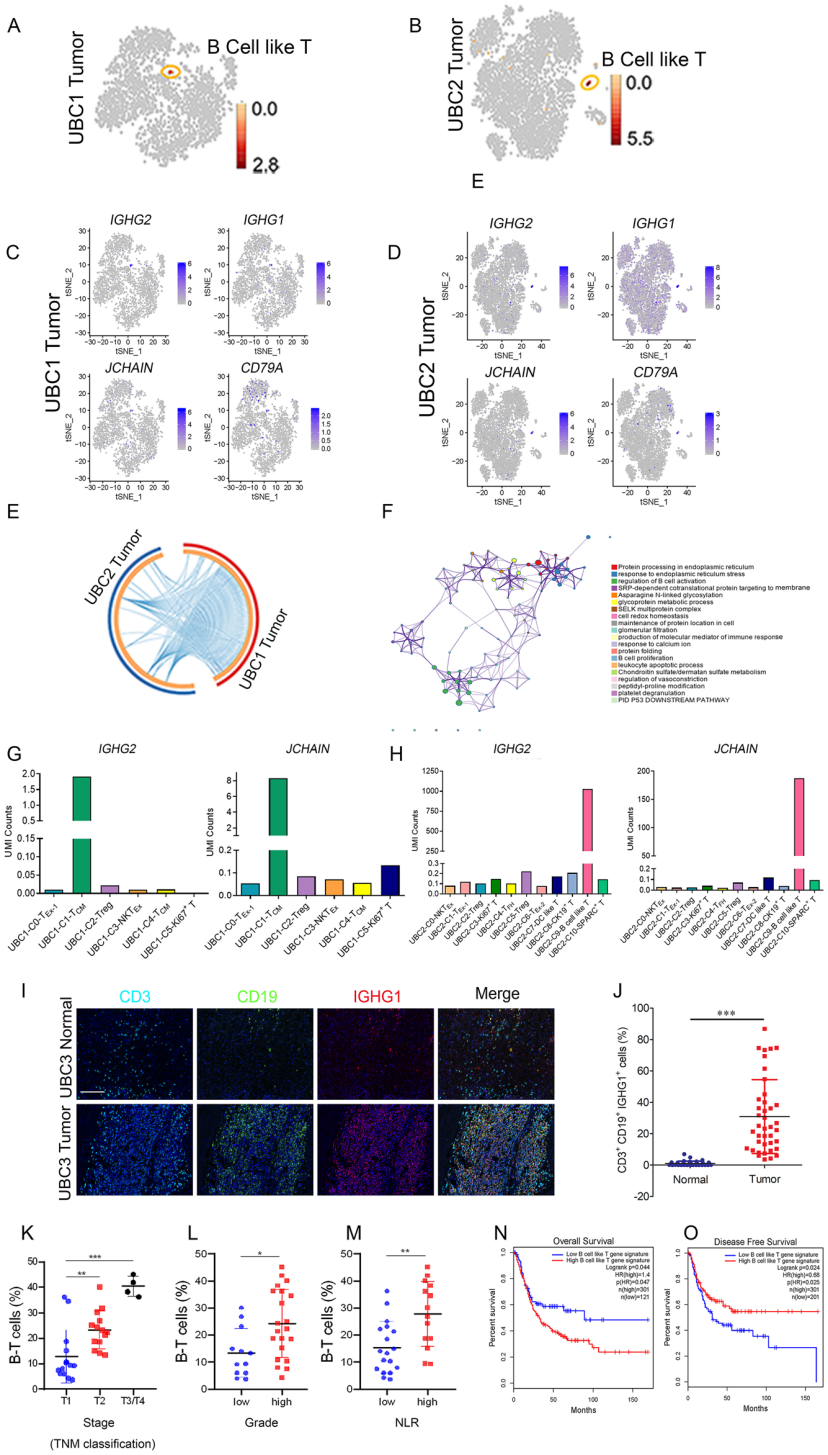
Characterization of B-T Cells and its clinical implication in UBC (**A**, **B**) The t-SNE projection of B-T cells in tumor tissues of UBC1 and UBC2, showing in red. (**C**–**D**) Expression levels of *IGHG1*, *IGHG2*, *JCHAIN* and *CD79A*, across 7939 (UBC1 2,446 and UBC2 5493) single T cells illustrated in t-SNE plots. (**E**) The gene expression status between tumor tissues of UBC1 and UBC2 were visualized in a circular layout via the Circos analysis tool. The red line of the outer circle manifests UBC1, and the blue one stands for UBC2. The orange line of the inner circle manifests the common genes shared by both UBC1 and UBC2. The light orange line in the circle indicates the functional correlation between genes. (**F**) Pathway enrichment of 193 (UBC1 86, UBC2 107) signature genes in B-T cells. (**G**) UMI counts of *IGHG2* and *JCHAIN* in C0-T_Ex_, C1-T_CM_, C2-Treg, C3-NKT_Ex_, C4-T_CM_ and C5-Ki67^+^ T clusters in UBC1. (**H**) UMI counts of *IGHG2* and *JCHAIN* in C0-NKT_Ex_, C1-T_Ex_-1, C2-Treg, C3-Ki67^+^ T, C4-T_FH_, C5-Treg, C6-T_Ex_-2, C7-DC like T, C8-CK19^+^ T, C9-B-T and C10-SPARC^+^ T clusters in UBC2. (**I**) Representative immunofluorescent staining with anti-CD3, CD19 and IGHG1 antibodies in NBT and UBC tissues. Bar = 125 μm. (**J**) The parentage of CD3^+^CD18^+^IGHG1^+^ B-T cells in NBT and UBC tissues. (**K**) Correlation analysis between staging and B-T cells in UBC patients. (**L**) Correlation analysis between grading and B- T cells in UBC patients. (**M**) Correlation analysis between NLR and B-T cells in UBC patients. (**N**–**O**) Kaplan–Meier curves comparing the OS and DFS between UBC patients expressing high or low levels of gene signature in B cell like T cells, log-rank test. n, patient number. Data are presented as mean ± SD. *P < 0.05, **P < 0.01.

A specific cluster named B-T cells existed in both UBC1 and UBC2 patients (Fig. 7A,B). Specifically, 86 and 107 differentially expressed genes were identified in B-T cells from UBC1 and UBC2, respectively (Supplementary Table 17). B-T cells expressed high level of immune checkpoints such as *GHG2*, *IGHG1*, *JCHAIN* and *CD79A* (Fig. 7C,D), and there was a high overlapping rate of specific genes among them (Fig. 7E). Moreover, the differentially expressed genes were involved in activation of B cell, B cell proliferation, induction of immune response, etc. (Fig. 7F), suggesting that B-T cells was actively involved in proliferation and antibodies production. However, the functional relations between these genes of B-T cells from UBC1 and UBC2 were rather diminutive (Supplementary Fig. 11), indicating the high heterogeneity of B-T cells. Furthermore, this cell cluster had increased expression of *IGHG2*, *IGHG1*, *JCHAIN* and *CD79A* (Fig. 7C,D,G,H) and CD3^+^CD19^+^IGHG1^+^ T cells significantly infiltrated in UBC (30.88%) compared to NBT (0.98%) (Fig. 7I,J).

Compared with T1 stage, the proportion of B-T cells in T2 (*P* < 0.01) and T3/T4 (*P* < 0.001) stages significantly increased (Fig. 7K). Similarly, the proportion of B-T cells significantly increased in UBC patients with higher grades (*P* < 0.05) and NLR (*P* < 0.01) (Fig. 7L,M). More importantly, UBC patients acquiring high expression of B-T cells signature genes (Supplementary Table 18) had a decreased OS as well as DFS compared to those of UBC patients acquiring low expression of these signature genes (Fig. 7N,O). Additionally, LIHC, uveal melanoma (UVM), LGG and ACC patients acquiring high expression of B-T cells signature genes had a decreased OS and DFS as compared to those of tumor patients acquiring low expression of these signature genes (Supplementary Fig. 12). In short, B-T cells were exclusively identified in UBC, which highly expressed *IGHG2*, *IGHG1*, *JCHAIN* and *CD79A*. The gene expression signature of B-T cells was associated with poor prognosis of patients with UBC and other tumor types.

## Discussion

UBC is characterized by a high rate of recurrence and has limited therapeutic options. Tumor-infiltrating T cells are of high heterogeneity in the tumor tissues, whose composition and numbers are closely related to the prognosis and therapeutic effects of UBC treatment. However, the composition and function of tumor-infiltrating T cells in UBC remain unclear. Here, scRNA-seq of the T cell populations in PBMC, NBT and tumor tissue from two UBC patients were performed. The transcriptional profiles of 30, 905 individual T cells provided a deep interrogation of phenotype and functional heterogeneity among tumor resident immune cell populations. We also identified four clusters, T_Ex_, NKT_Ex_, Ki67^+^ and B-T subtypes, were significantly enriched in UBC, which correlated with the poor prognosis of UBC.

In our study, T_N_, T_TN_, T_Eff_, NKT, Treg and DC like T cell subtypes were mainly enriched in the PBMC and NBT of UBC patients. T_Eff_ highly expressed effector molecules-related genes such as *CMC1*, and NKT cells expressed NK cell surface markers and effector molecules-related genes, such as *IFNG*, *GZMB* and *GZMA*, suggesting the potential of effector functions. There were T_N_, T_TN_, T_CM_ in PBMC, indicating the different developmental stages of T cell population, suggesting the differentiation process and functional activation in T cell population of PBMC.

T_Ex_ is a exhausted subset of T cells that specifically overexpressing *IKZF3, LUC7L3, TRGC2*, *LAG3*, and *TIM3,* which were mainly involved in T cell development, antigen recognition, and expression of heterogeneous receptors. It is particularly noteworthy that LAG3 and TIM3 are genes that express T cell inhibitory receptors and negatively regulate the killing ability of T cells. By blocking the signal reception of inhibitory receptors, the exhaustion state of T cells could be partially alleviated, thereby enhancing their ability to kill tumors. Compared with T cells in PBMC and NBT, T_Ex_ cells were significantly enriched in UBC and highly expressed immune checkpoints encoding genes, which was similar to the T_Ex_ cells in liver cancer^15^, breast cancer^14^ and non-small-cell lung cancer^13^. Additionally, cytokine granulocyte-macrophage colony stimulating factor (GM-SCF) encoding gene *CSF2* and chemokine CCL5 encoding gene *CCL5* were specifically expressed in T_Ex_ cells, which recruted Treg cells into tumor microenvironment and promoted tumor growth^27^. Furthermore, *IKZF3* and *TRGC2* were also highly expressed in T_Ex_ and CD8^+^PD1^+^TIM3^+^TRGC2^+^ T_Ex_ accounts for 3.47% of CD3^+^ T cells in UBC, signifying that *IKZF3* and *TRGC2* are potential biomarkers of T_Ex_. More importantly, the gene expression signature of T_Ex_ cells remarkably associated with poor prognosis of UBC, LUSC, LIHC, LGG and ACC. In recent years, using the autoimmune system to fight against tumor cells has become an important approach in tumor immunotherapy. A variety of treatment methods have been explored for different targets of reversing the process of T cell exhausted, such as immunocheckpoint inhibitor treatment, epigenetics treatment, CAR-T adoptive cellular immunotherapy and other programs. Some studies have shown that the combination of inhibitory receptors, such as the use of anti PD-1/PD-L1 and anti CTLA-4 antibodies, can significantly reverse the exhaustion of T cells and enhance the immune response of various tumor patients^28^. In the CT26 colon adenocarcinoma model^29^, the combination of anti TIM-3 and anti PD-1 drugs is more effective than anti PD-1 therapy alone. Guo et al.^30^ pointed out that the combination of anti TIM-3 and anti CD137/4-1BB antibodies can also significantly inhibit the growth of mouse ovarian tumors. Thus, T_Ex_ cells and its biomarkers were the promising targets for cancer immunotherapy and render the development of robust and effective immunotherapy strategies towards UBC.

The killing ability of T cells mainly depends on antigen presentation. Unlike T cells, NKT cell can kill without contacting antigens. However, if NKT cell are exposed to antigens for a long time, they will also be in a state of exhaustion like T cells. NKT_Ex_ cells specifically overexpresses *ATF3, CSF2, NR4A1, LAG3*, and *TIM3*, which are mainly involved in transcriptional activation, regulation of lymphocyte production, and expression of heterogeneous receptors. We also observed a higher distribution of NKT_Ex_ (CD8^+^CD56^+^PD1^+^TIM3) in UBC, which accounts for 16.92% of CD3^+^ T cells in UBC. NKT_Ex_ cells expressed high level of immune checkpoint encoding genes and exhausted-related genes, which was prone to be an exhaustive state and similar to the NKT_Ex_ cells in pancreatic cancer^12^. Previous studies indicated that NKG2A targeting with monalizumab is a novel checkpoint inhibitory mechanism promoting anti-lymphoma immunity by enhancing the activity of both T and NK cells^31^. Here, we also found that *KLRC1* (encoding NKG2A) was highly expressed in both T_Ex_ and NKT_Ex_ cells  of UBC, which could be a promising target. Furthermore, *ATF3* and *NR4A1* are potential biomarkers of NKT_Ex_ cells. More importantly, not only UBC patients, but also LUSC, LIHC, LGG and ACC ones acquiring high expression of NKT_Ex_ cells signature genes had a decreased OS as compared to those of tumor patients acquiring low expression of these signature genes. As is well known, immune checkpoints play an important role in the production of immunosuppressive tumor microenvironment, leading to NK cell failure and tumor immune escape. Therefore, NK cells must reverse their dysfunctional state and increase their effector effect to improve the efficiency of cancer immunotherapy. Blocking immune checkpoints not only saves NK cells from failure, but also enhances their powerful anti-tumor activity^32^. In melanoma, fibrosarcoma, colon cancer and leukemia models, the co-blocking of TIM-3 and PD-1 showed higher anti-tumor efficacy, more complete tumor regression and longer survival period^29,33,34^. Besides, the combination of NKG2A monoclonal antibody or TIGIT monoclonal antibody plus PD-1 monoclonal antibody or cetuximab showed encouraging results in the treatment of patients with advanced solid tumors^31,35^. Thus, NKT_Ex_ cells and its biomarkers were the promising candidates for UBC immunotherapy.

Ki67^+^ T cells were identified in the UBC patients for the first time and accounted for 11.85% of CD3^+^ T cells in UBC. *MKI67*, *AURKA*, *TOP2A* and *UBE2C* were highly expressed in Ki67^+^ T cells, indicating an active proliferation status and potential contribution to the occurrence, development, and metastasis of UBC. Ki67^+^ T cell subsets were previously identified in colorectal cancer^36^ and multiple myeloma^37^. Moreover, the percentage of Ki67^+^ lymphocytes was significantly higher in patients with multiple myeloma and monoclonal gammopathy compared with the normal controls, which was associated with disease stage. Here, we found that the gene expression signature of Ki67^+^ T cells remarkably associated with poor prognosis of UBC, LUSC, LIHC, LGG and ACC. Therefore, Ki67^+^T cells are potential candidate cells for UBC immunotherapy, and further research is needed on their application in immunotherapy in the future.

Finally, we identified another novel and specific subpopulation, namely B-T cells, in UBC. Previous studies suggested that the proportion of B-T cells was high in cancer compared with that of controls, such as esophageal cancer, non-Hodgkin's lymphoma, lung cancer, breast cancer, liver cancer, etc.^38^. Gene expression profiling revealed that this cell cluster had increased expression of *IGHG2*, *IGHG1*, *JCHAIN* and *CD79A*, These genes are mainly involved in the coding of immunoglobulins and the formation of proteins, indicating the B cell properties in T cell populations. Besides, it also had the expression of *CD3*, suggesting the existence of matured T cell population. More importantly, not only UBC patients, but also LIHC, UVM, LGG and ACC ones acquiring high expression of signature genes of B-T cells had a decreased OS and DFS as compared to those of tumor patients acquiring low expression of these signature genes. Thus, B-T cells were a potential candidate for UBC immunotherapy, whose phenotype and role need further investigation.

In UBC, T cell exhaustion might result from long-duration antigen exposures and continuous inflammation^39^. The cell-to-cell signals including prolonged T cell receptor (TCR) engagement and co-stimulatory and/or co-inhibitory signals, inflammatory cytokines and suppressive cytokines, and tissue and microenvironmental influences are the potential reasons of T cell exhaustion^40^. In this study, exhausted T or NKT cells may be converted by effector CD8^+^ T cells^41,42^. Mechanically, persistent antigen exposure, the elevated expression of inhibitory receptors (*TIM3, PDCD1, LAG3, CTLA4, IKZF3* and *TRGC2*), surface markers (*CD3, CD4, CD8, CD19* and *CD56*), transcrition factors (*NR4A1, ATF3*), immunosuppressive cytokines (*VEGF, IL10* and *IL4*) are the potential reasons of T cell exhaustion in UBC. Although Ki67^+^ T cells and B cell like T cells were previously reported in colorectal cancer and multiple myeloma, esophageal cancer, non-Hodgkin's lymphoma, lung cancer, breast cancer, liver cancer, etc.. However, the causes of these increase have not been clearly described, which need further investigation.

In short, this article conducts scRNA-seq from T cells derived from tumor tissue, normal tissue adjacent to tumor and PBMC of two UBC patients, and more UBC cases with different pathological characteristics were needed to better understand the heterogeneity of T cell subsets in UBC microenvironment. In addition, a large number of literatures have shown that T cell function exhaustion is an important mechanism of tumor immune escape^40^. The functional assay to investigate immune-suppressive activity of T_Ex_ cells, NKT_Ex_ cells, Ki67^+^ T cells and B-T cells could be carried out in the future to reveal the relationship between the major T cell subtypes and immune escape.

## Conclusions

In this study, scRNA-seq was applied for the first time to provide a thorough description of T cells in PBMC, NBT and tumor tissues of UBC patients, and describing gene expression characteristics and potential functions. The heterogeneity of T cell subsets in tumor microenvironment was revealed, which contributed to the immunosuppressive microenvironment. Notably, T_Ex_, NKT_Ex_, Ki67^+^ T and B-T cells were exclusively enriched in UBC, which were significantly associated with poor survival in patients with UBC and various tumor types. The identified T cells subsets and novel biomarkers provided a theoretical foundation for the development of effective tumor immunotherapies.

### Supplementary Information

Supplementary Figures.

Supplementary Table 1.

Supplementary Table 2.

Supplementary Table 3.

Supplementary Table 4.

Supplementary Table 5.

Supplementary Table 6.

Supplementary Table 7.

Supplementary Table 8.

Supplementary Table 9.

Supplementary Table 10.

Supplementary Table 11.

Supplementary Table 12.

Supplementary Table 13.

Supplementary Table 14.

Supplementary Table 15.

Supplementary Table 16.

Supplementary Table 17.

Supplementary Table 18.

## Data Availability

The raw sequence data reported in this paper have been deposited in the Genome Sequence Archive (Genomics, Proteomics & Bioinformatics 2021) in National Genomics Data Center (Nucleic Acids Res 2022), China National Center for Bioinformation/Beijing Institute of Genomics, Chinese Academy of Sciences (GSA-Human: HRA006379) that are publicly accessible at https://ngdc.cncb.ac.cn/gsa-human/submit/hra/submit.
