# Supplementary material for: Specific subsets of urothelial bladder carcinoma infiltrating T cells associated with poor prognosis
Source: Sci Rep. 2023 Aug 7;13:12801. doi: 10.1038/s41598-023-39208-0 (PMC10406853; doi:10.1038/s41598-023-39208-0)
Supplement: Supplementary file 1 — Supplementary Figures. [file 41598_2023_39208_MOESM1_ESM.docx]

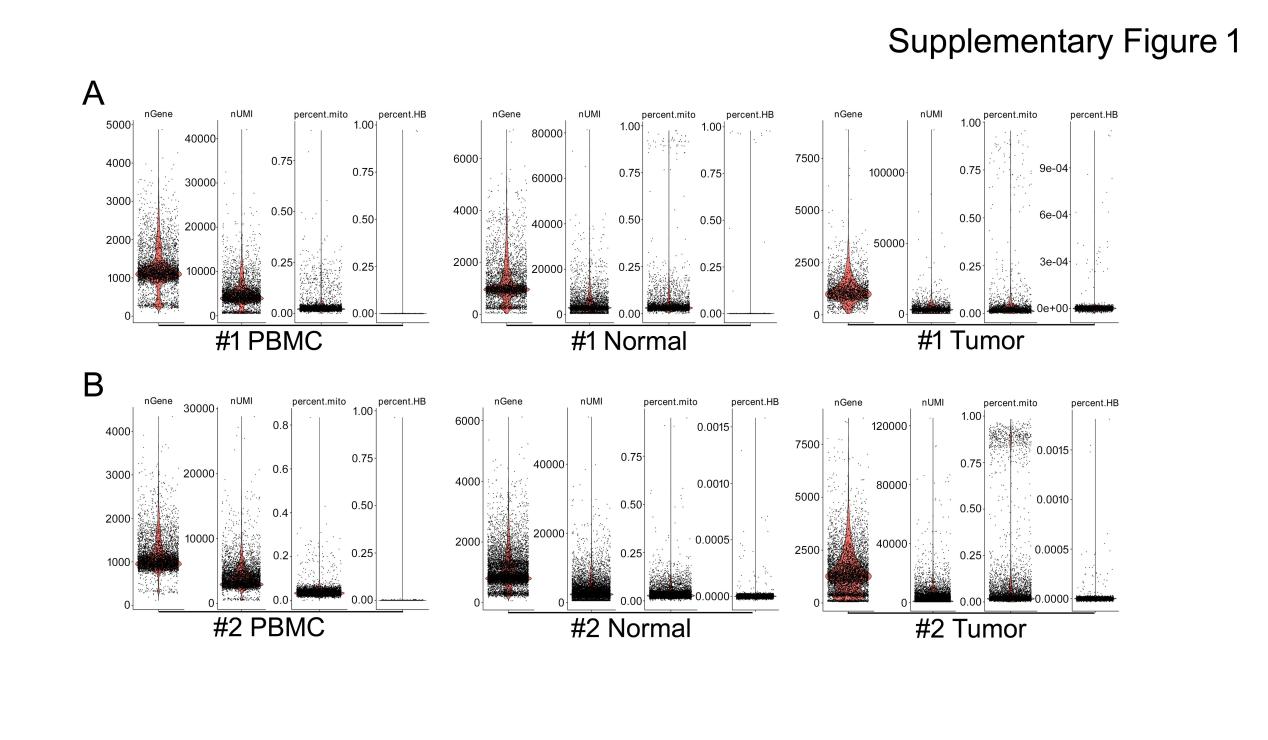


**Supplementary Figure 1.** The single-cell sequencing data and filteration parameters. (A and B) The number of genes detected, the number of UMI, the proportion of mitochondrial UMI and the proportion of erythrocyte UMI of BC1 and BC2.


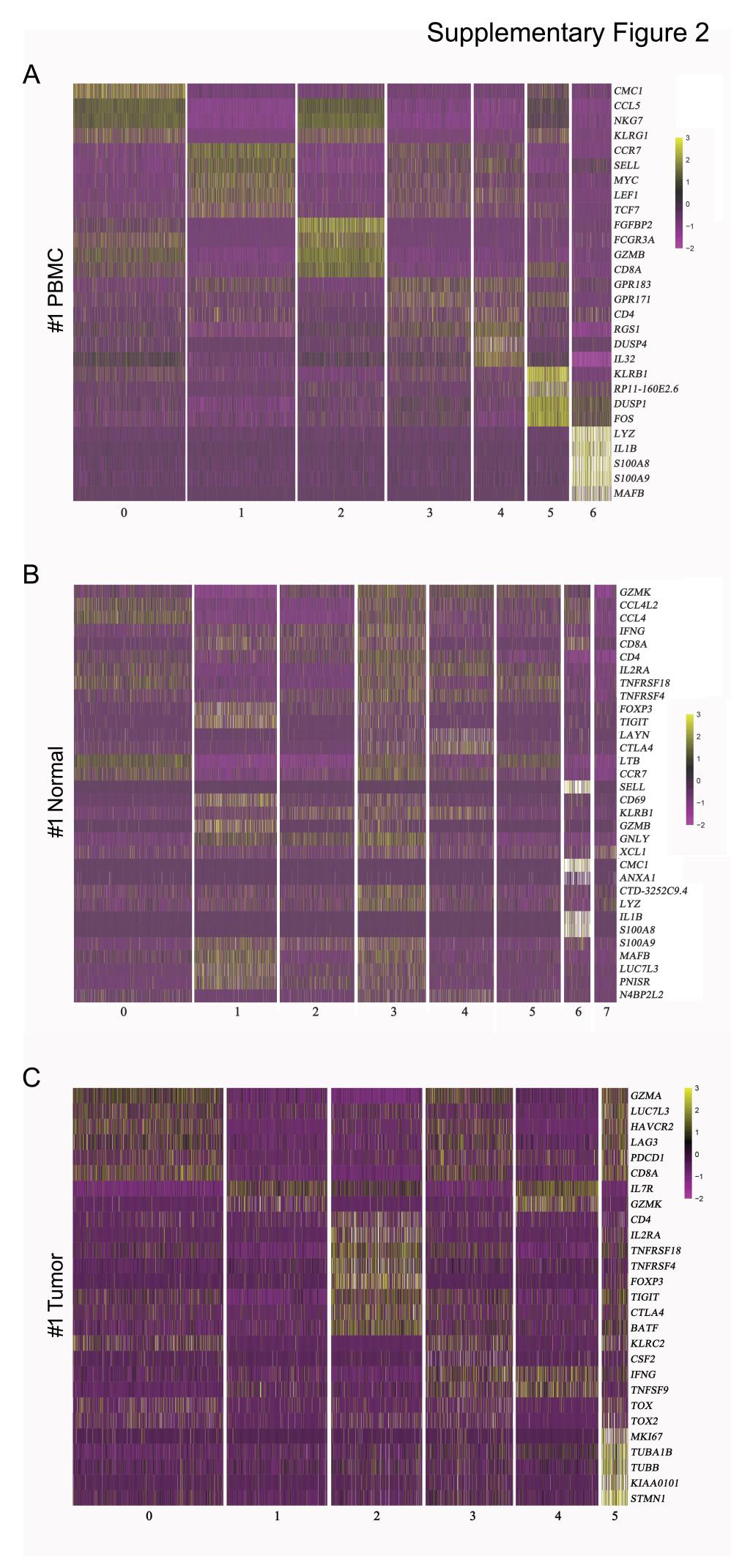


**Supplementary Figure 2.** Heat maps of T cell gene expression in BC1. (A-C) Heat map of T cell gene expression of PBMC, normal bladder tissue and tumor tissue from BC1.


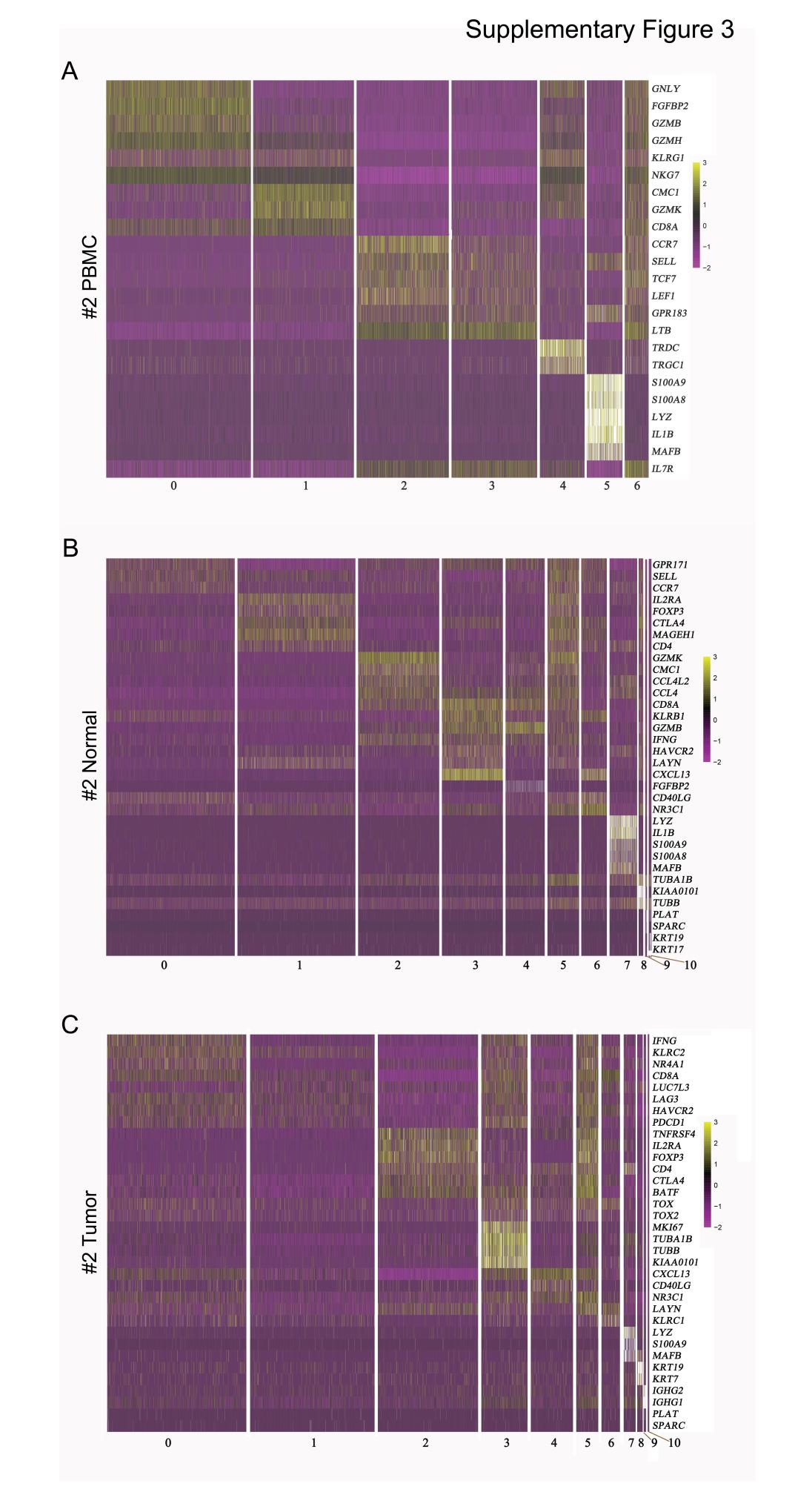


**Supplementary Figure 3.** Heat maps of T cell gene expression in BC2. (A-C) Heat map of T cell gene expression of PBMC, normal bladder tissue and tumor tissue from BC2.


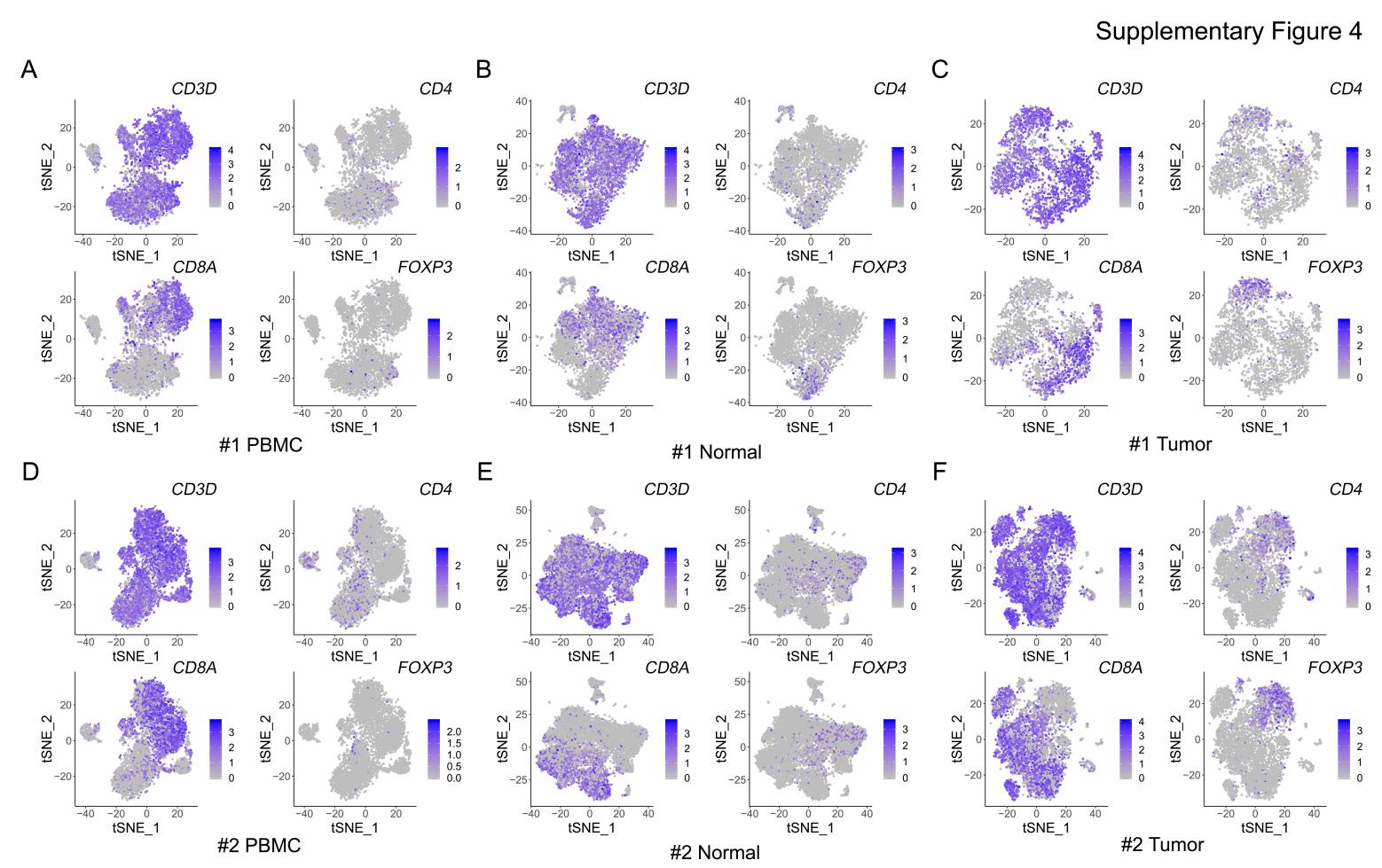


**Supplementary Figure 4**. Expression of *CD3D*, *CD4*, *CD8A*, and *FOXP3* in tumor-infiltrating T cells. (A-C). Expression of *CD3D*, *CD4*, *CD8A*, and *FOXP3* in tumor-infiltrating T cells of PBMC, normal bladder tissue and tumor tissue from BC1. (D-F). Expression of *CD3D*, *CD4*, *CD8A*, and *FOXP3* in tumor-infiltrating T cells of PBMC, normal bladder tissue and tumor tissue from BC2.

**
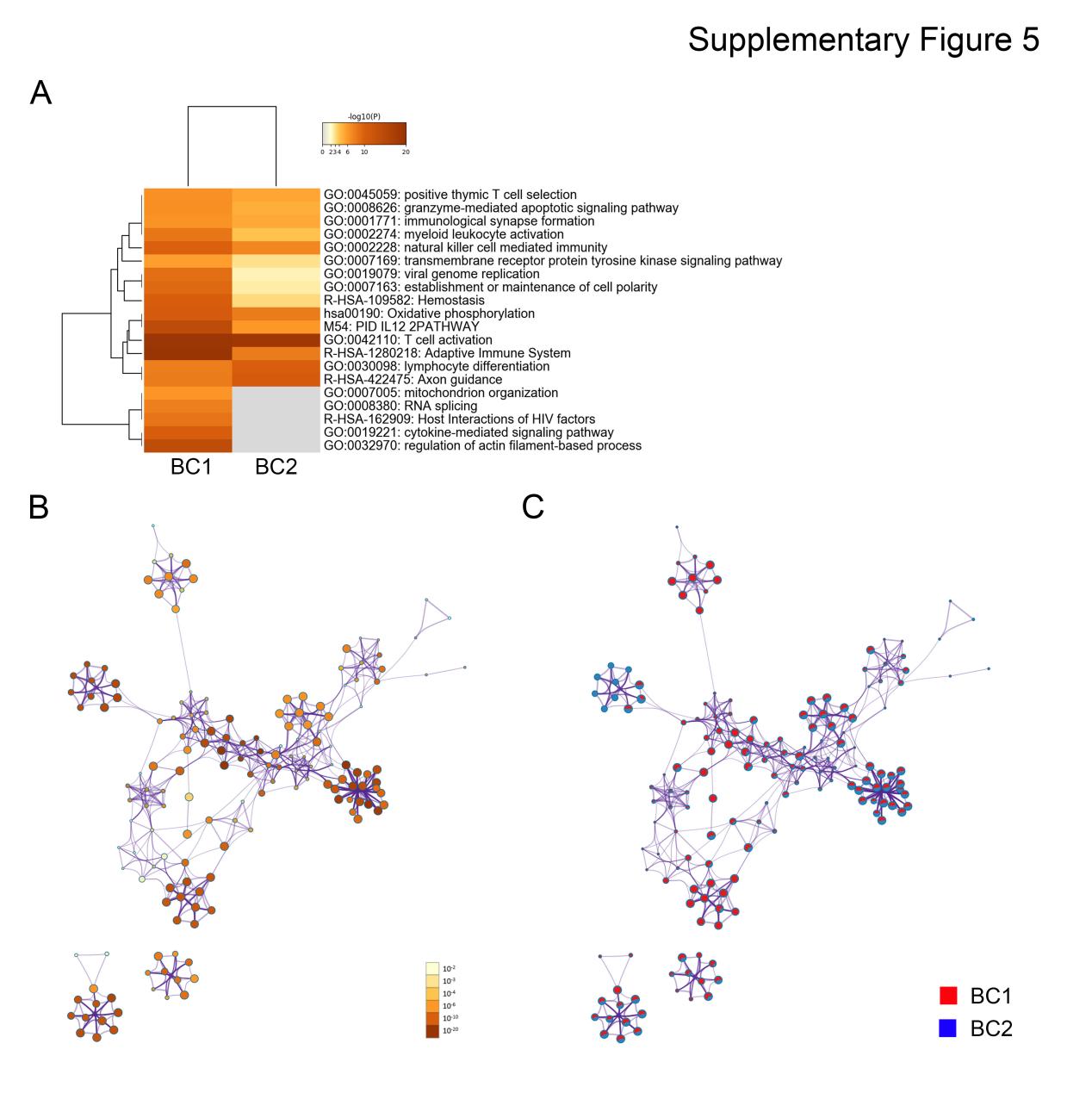
Supplementary Figure 5**. Pathway enrichment analysis of T_Ex_. (A) The Top 20 enrichment pathway of T_Ex_ in BC1 and BC2 were demonstrated by gene set pathway enrichment analyses, colored by p-values. (B) Functional Gene Networks derived from gene set pathway enrichment analyses shows the relationship between the pathways of T_Ex_. Each node represents an enrichment pathway, and nodes of the same color represent the same signaling pathway.


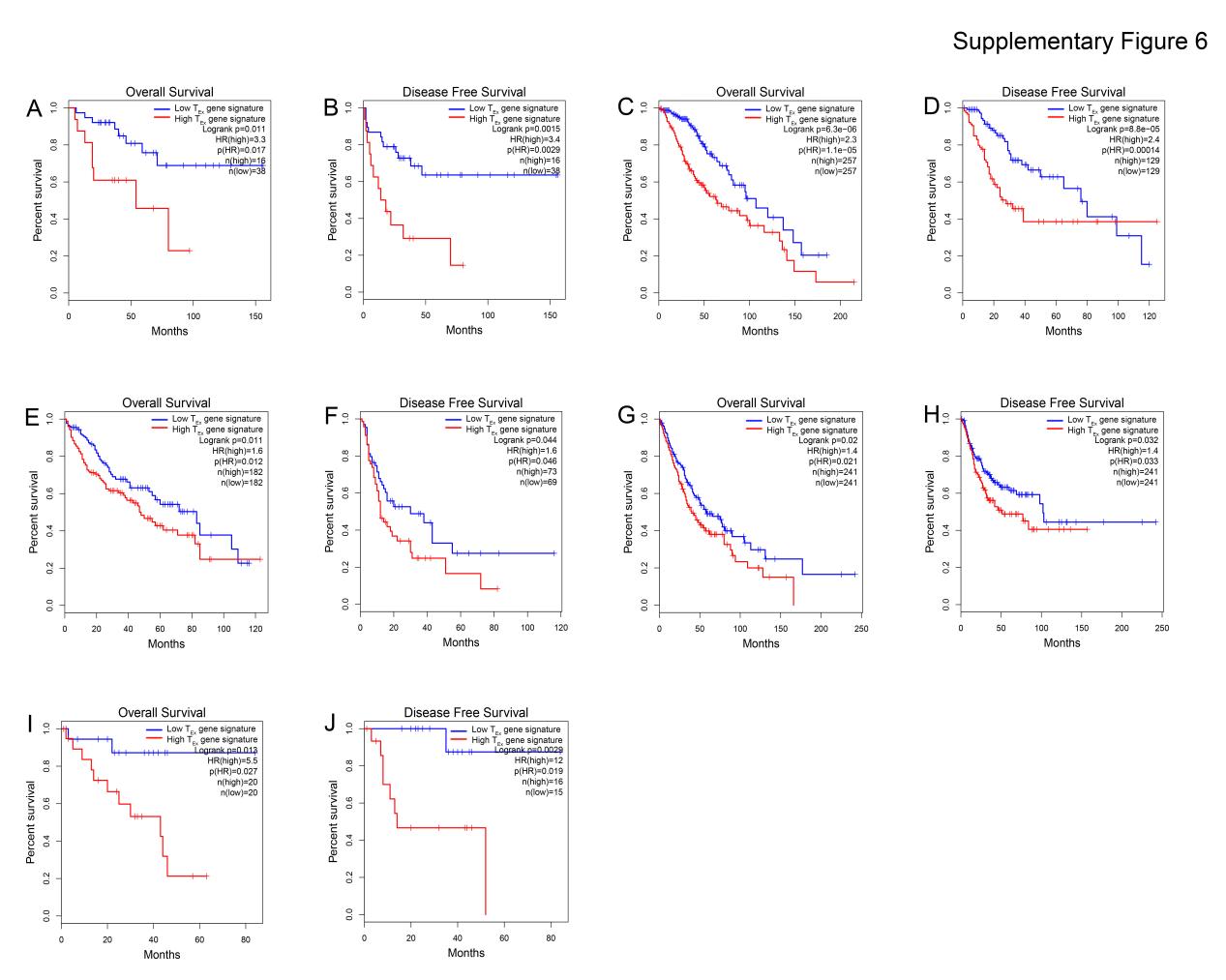


**Supplementary Figure 6**. Kaplan-Meier curves of the overall survival and disease-free survival of T_Ex_. (A-B) OS and DFS of LUAD patients expressing high or low levels of signature genes of T_Ex_. (C-D) OS and DFS of LUSC patients expressing high or low levels of signature genes of T_Ex_. (E-F) OS and DFS of LIHC patients expressing high or low levels of signature genes of T_Ex_. (G-H) OS and DFS of LGG patients expressing high or low levels of signature genes of T_Ex_. (I-J) OS and DFS of ACC patients expressing high or low levels of signature genes of T_Ex_. Kaplan-Meier curves comparing the overall survival and disease-free survival between patients of various tumor types expressing high or low levels of signature genes of T_Ex_, log-rank test. n, patient number. Data are presented as mean ± SD. P < 0.05; P < 0.01.

**
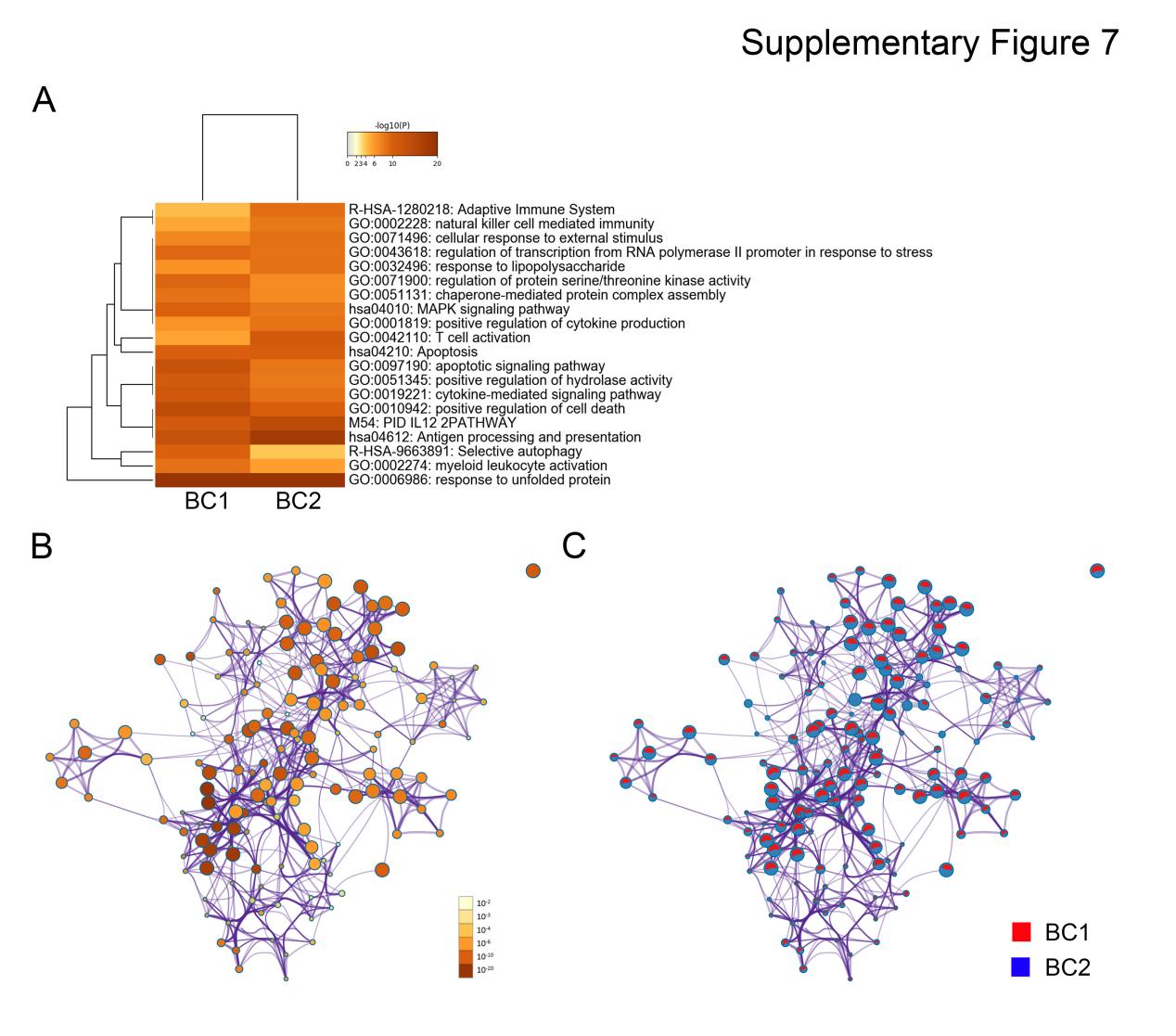
Supplementary Figure 7**. Pathway enrichment analysis of NKT_Ex_. (A) The Top 20 enrichment pathway of NKT_Ex_ in BC1 and BC2 were demonstrated by gene set pathway enrichment analyses, colored by p-values. (B) Functional Gene Networks derived from gene set pathway enrichment analyses shows the relationship between the pathways of NKT_Ex_. Each node represents an enrichment pathway, and nodes of the same color represent the same signaling pathway.

**
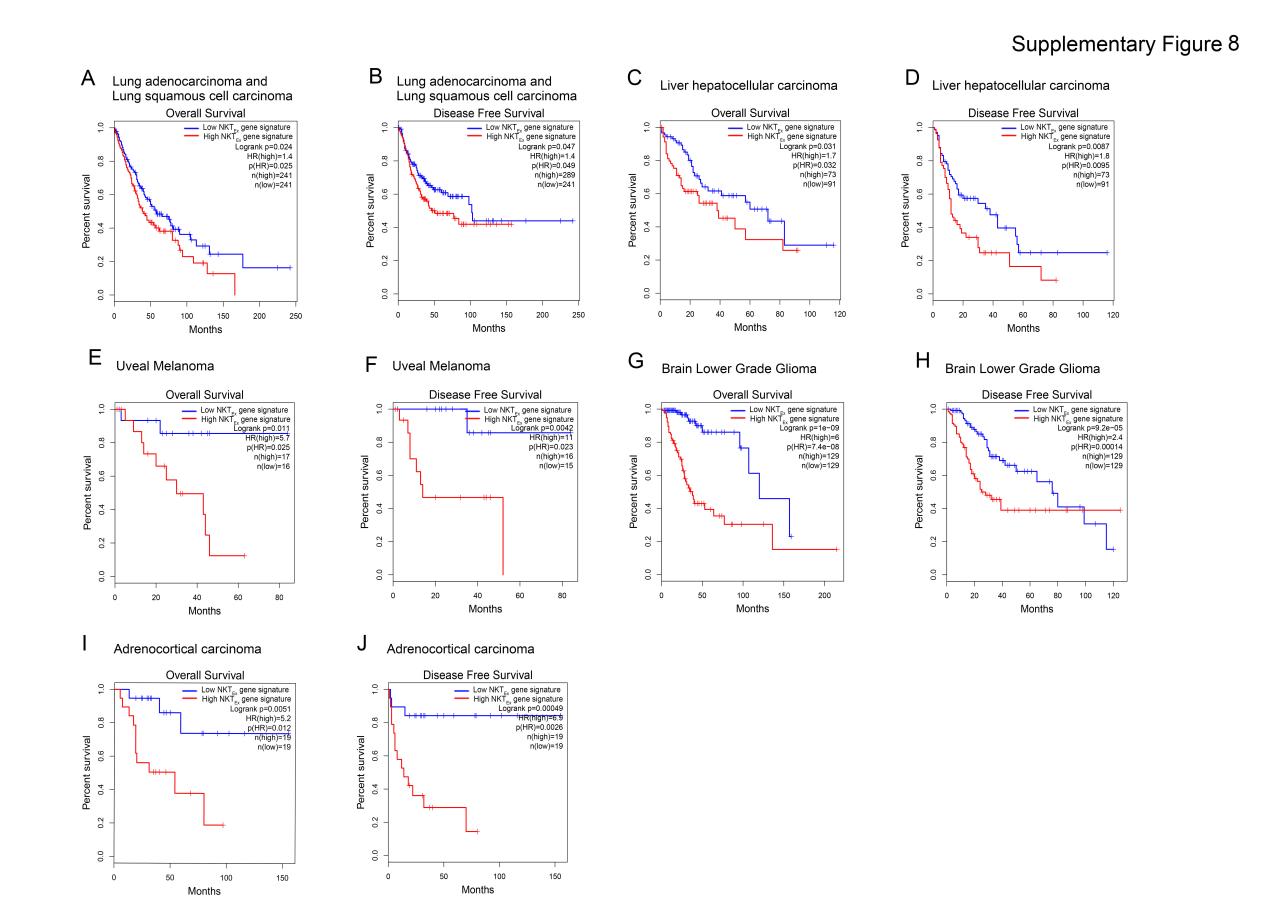
Supplementary Figure 8**. Kaplan-Meier curves of the overall survival and disease-free survival of NKT_Ex_. (A-B) OS and DFS of LUAD patients expressing high or low levels of signature genes of NKT_Ex_. (C-D) OS and DFS of LUSC patients expressing high or low levels of signature genes of NKT_Ex_. (E-F) OS and DFS of LIHC patients expressing high or low levels of signature genes of NKT_Ex_. (G-H) OS and DFS of LGG patients expressing high or low levels of signature genes of NKT_Ex_. (I-J) OS and DFS of ACC patients expressing high or low levels of signature genes of NKT_Ex_. Kaplan-Meier curves comparing the overall survival and disease-free survival between patients of various tumor types expressing high or low levels of signature genes of NKT_Ex_, log-rank test. n, patient number. Data are presented as mean ± SD. P < 0.05; P < 0.01.

**
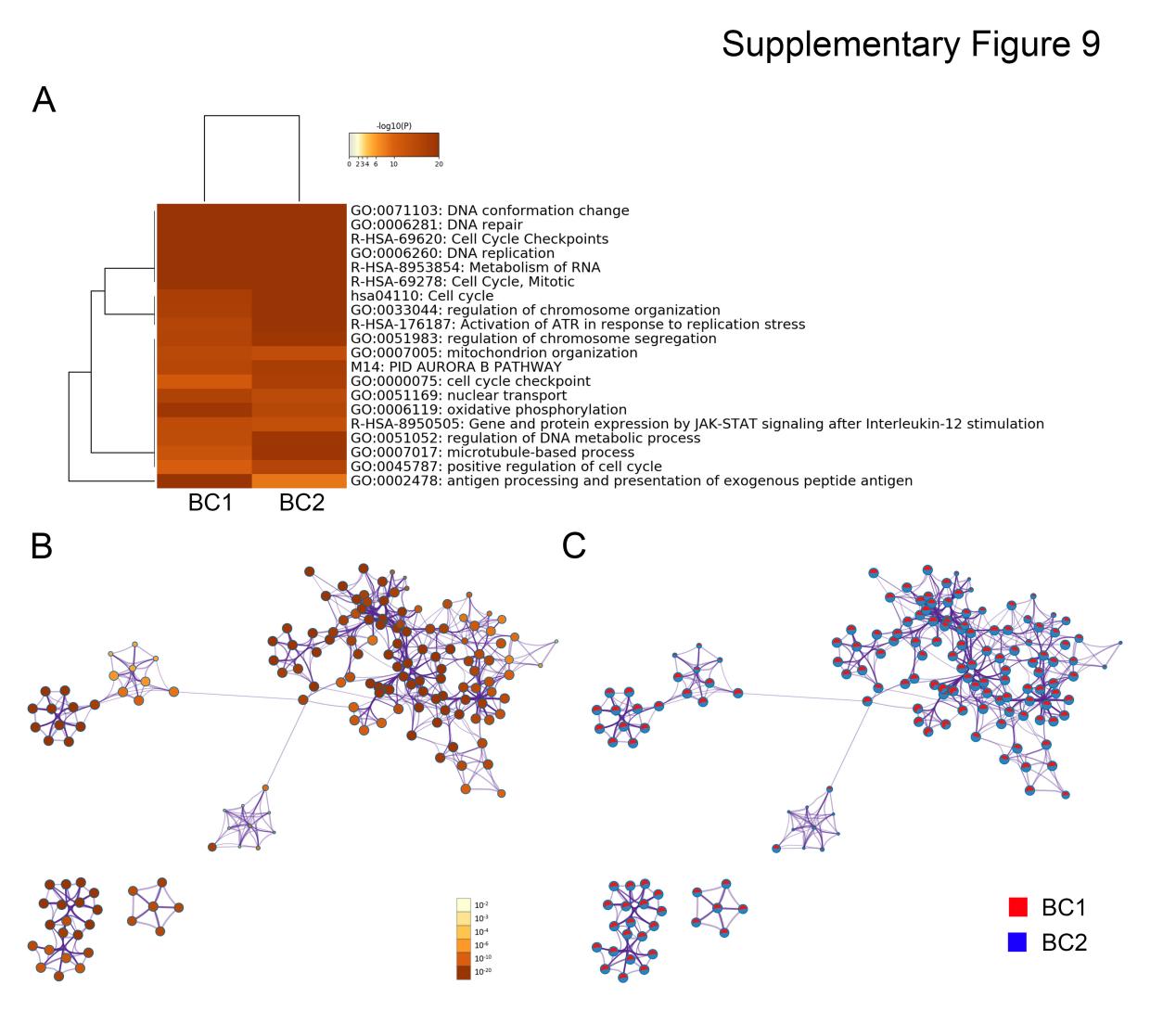
Supplementary Figure 9**. Pathway enrichment analysis of KI67^+^ T cells. (A) The Top 20 enrichment pathway of KI67^+^ T cells in BC1 and BC2 were demonstrated by gene set pathway enrichment analyses, colored by p-values. (B) Functional Gene Networks derived from gene set pathway enrichment analyses shows the relationship between the pathways of KI67^+^ T cells. Each node represents an enrichment pathway, and nodes of the same color represent the same signaling pathway.


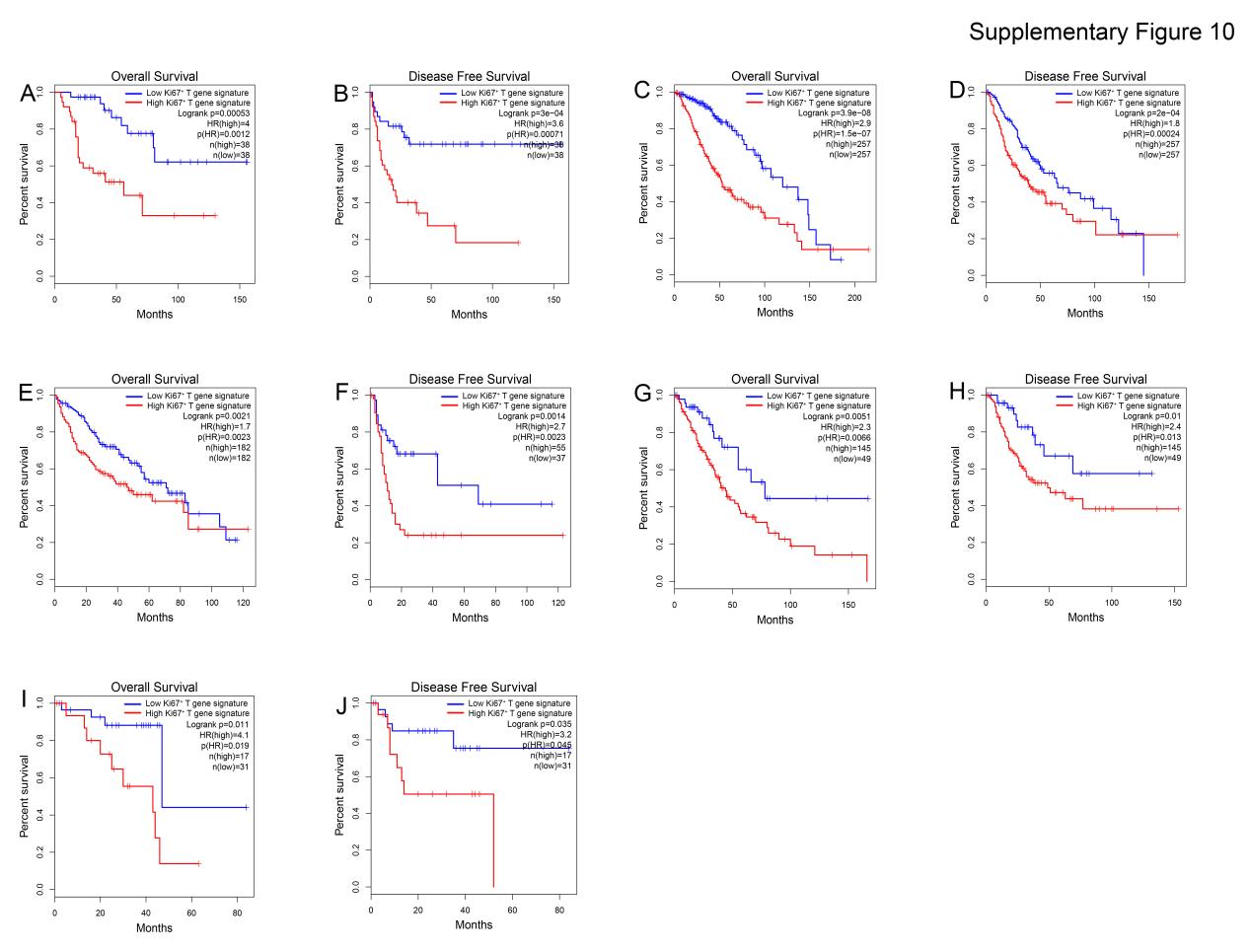


**Supplementary Figure 10**. Kaplan-Meier curves of the overall survival and disease-free survival of KI67^+^ T cells. (A-B) OS and DFS of LUAD patients expressing high or low levels of signature genes of KI67^+^ T cells. (C-D) OS and DFS of LUSC patients expressing high or low levels of signature genes of KI67^+^ T cells. (E-F) OS and DFS of LIHC patients expressing high or low levels of signature genes of KI67^+^ T cells. (G-H) OS and DFS of LGG patients expressing high or low levels of signature genes of KI67^+^ T cells. (I-J) OS and DFS of ACC patients expressing high or low levels of signature genes of KI67^+^ T cells. Kaplan-Meier curves comparing the overall survival and disease-free survival between patients of various tumor types expressing high or low levels of signature genes of KI67^+^ T cells, log-rank test. n, patient number. Data are presented as mean ± SD. P < 0.05; P < 0.01.

**
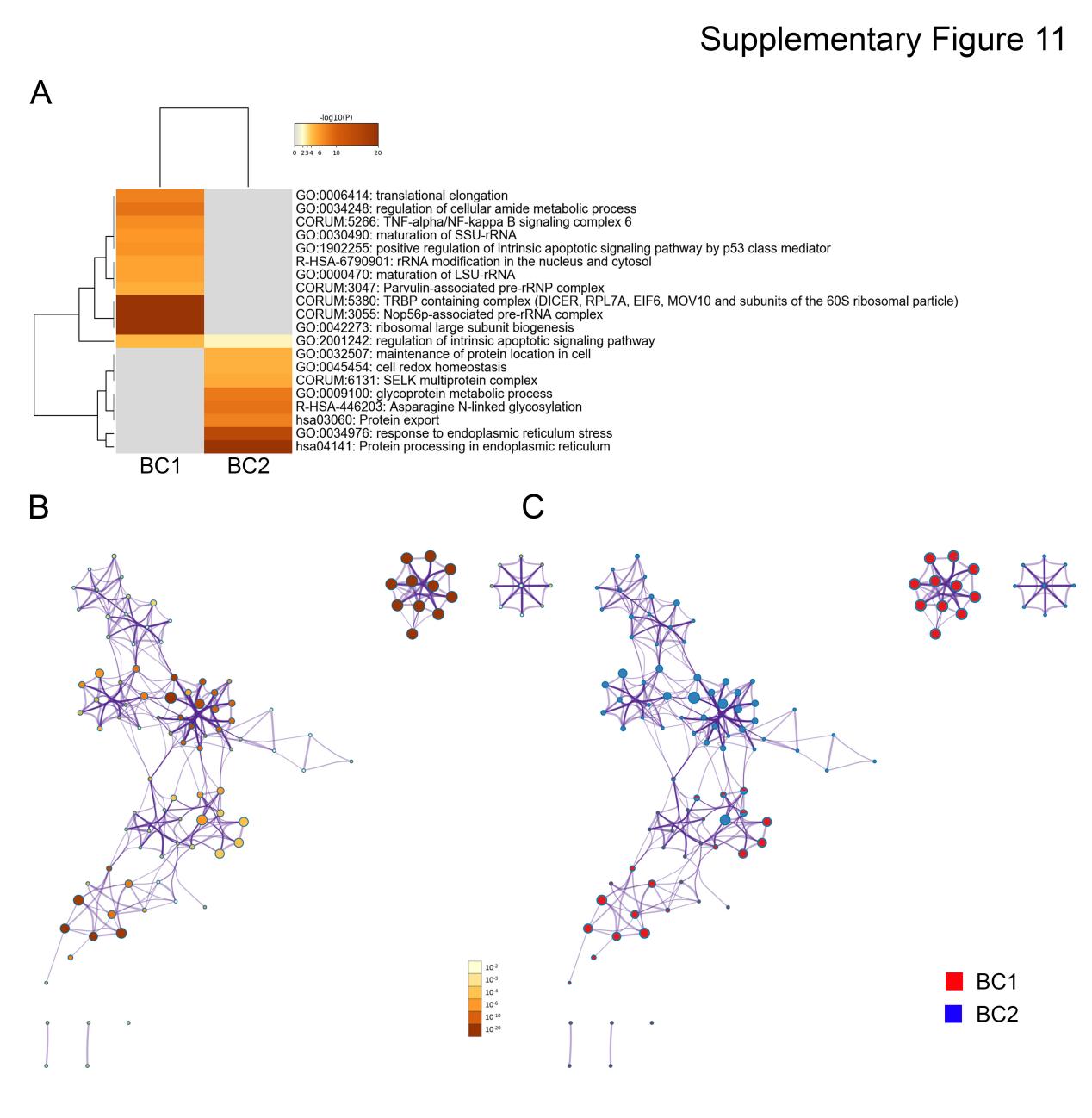
Supplementary Figure 11**. Pathway enrichment analysis of B cell like T cells (A) The Top 20 enrichment pathway of B cell like T cells in BC1 and BC2 were demonstrated by gene set pathway enrichment analyses, colored by p-values. (B) Functional Gene Networks derived from gene set pathway enrichment analyses shows the relationship between the pathways of B cell like T cells. Each node represents an enrichment pathway, and nodes of the same color represent the same signaling pathway.


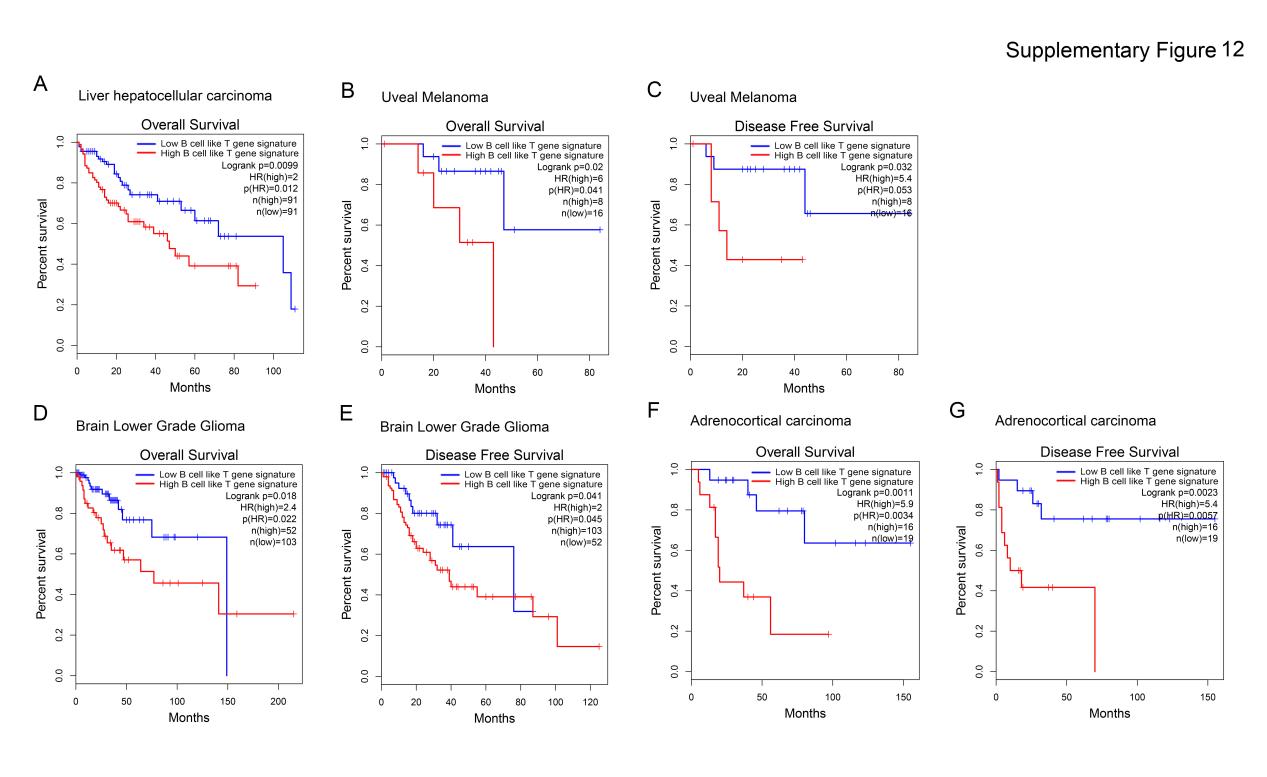


**Supplementary Figure 12**. Kaplan-Meier curves of the overall survival and disease-free survival of B-T cells. (A) OS of LIHC patients expressing high or low levels of signature genes of B-T T cells. (B-C) OS and DFS of UVM patients expressing high or low levels of signature genes of B-T T cells. (D-E) OS and DFS of LGG patients expressing high or low levels of signature genes of B-T T cells. (F-G) OS and DFS of ACC patients expressing high or low levels of signature genes of B-T T cells. Kaplan-Meier curves comparing the overall survival and disease-free survival between patients of various tumor types expressing high or low levels of signature genes of B-T T cells, log-rank test. n, patient number. Data are presented as mean ± SD. P < 0.05; P < 0.01.
